# Therapeutic potential of allosteric HECT E3 ligase inhibition

**DOI:** 10.1016/j.cell.2025.03.001

**Published:** 2025-05-15

**Authors:** Alexander M.K. Rothman, Amir Florentin, Florence Zink, Catherine Quigley, Olivier Bonneau, Rene Hemmig, Amanda Hachey, Tomas Rejtar, Maulik Thaker, Rishi Jain, Shih-Min Huang, Daniel Sutton, Jan Roger, Ji-Hu Zhang, Sven Weiler, Simona Cotesta, Johannes Ottl, Salil Srivastava, Alina Kordonsky, Reut Avishid, Elon Yariv, Ritu Rathi, Oshrit Khvalevsky, Thomas Troxler, Sarah K. Binmahfooz, Oded Kleifeld, Nicholas W. Morrell, Marc Humbert, Matthew J. Thomas, Gabor Jarai, Rohan E.J. Beckwith, Jennifer S. Cobb, Nichola Smith, Nils Ostermann, John Tallarico, Duncan Shaw, Sabine Guth-Gundel, Gali Prag, David J. Rowlands

**Affiliations:** 1 University of Sheffield, Sheffield, UK; 2 Novartis BioMedical Research (NBR), Cambridge, MA, USA; 3 School of Neurobiology, Biochemistry and Biophysics, The Life Sciences Faculty, Tel Aviv University, Tel Aviv, Israel; 4 Novartis BioMedical Research (NBR), Basel, Switzerland; 5 Novartis Institutes for BioMedical Research (NIBR), Horsham, UK; 6 Faculty of Biology, Technion—Israel Institute of Technology, Haifa 32000, Israel; 7 Department of Medicine, School of Clinical Medicine, University of Cambridge, Cambridge, UK; 8 Université Paris-Saclay, INSERM UMR_S 999 (HPPIT), Service de Pneumologie et Soins Intensifs Respiratoires, Hôpital Bicêtre (Assistance Publique Hôpitaux de Paris), Le Kremlin Bicêtre, France; 9 Sagol School of Neuroscience, Tel Aviv University, Tel Aviv, Israel; 10 Department of Clinical Biochemistry, Faculty of Medicine, King Abdulaziz University, Jeddah, Saudi Arabia

**Keywords:** ubiquitin E3 ligase, pulmonary arterial hypertension, SMURF1, E6AP, HECT, allosteric inhibition, small molecule, glycine hinge, vascular remodeling, drug discovery

## Abstract

Targeting ubiquitin E3 ligases is therapeutically attractive; however, the absence of an active-site pocket impedes computational approaches for identifying inhibitors. In a large, unbiased biochemical screen, we discover inhibitors that bind a cryptic cavity distant from the catalytic cysteine of the homologous to E6-associated protein C terminus domain (HECT) E3 ligase, SMAD ubiquitin regulatory factor 1 (SMURF1). Structural and biochemical analyses and engineered escape mutants revealed that these inhibitors restrict an essential catalytic motion by extending an α helix over a conserved glycine hinge. SMURF1 levels are increased in pulmonary arterial hypertension (PAH), a disease caused by mutation of bone morphogenetic protein receptor-2 (BMPR2). We demonstrated that SMURF1 inhibition prevented BMPR2 ubiquitylation, normalized bone morphogenetic protein (BMP) signaling, restored pulmonary vascular cell homeostasis, and reversed pathology in established experimental PAH. Leveraging this deep mechanistic understanding, we undertook an *in silico* machine-learning-based screen to identify inhibitors of the prototypic HECT E6AP and confirmed glycine-hinge-dependent allosteric activity *in vitro*. Inhibiting HECTs and other glycine-hinge proteins opens a new druggable space.

## Highlights

• A high-throughput screen reveals specific inhibitors of the HECT E3 ligase SMURF1
• The allosteric inhibitor limits an essential motion of a conserved glycine hinge
• The inhibitor reverses established pulmonary hypertension in animal models
• Allosteric mechanism-based *in silico* screening identifies inhibitors of E6AP

## Graphical abstract

**Figure:**
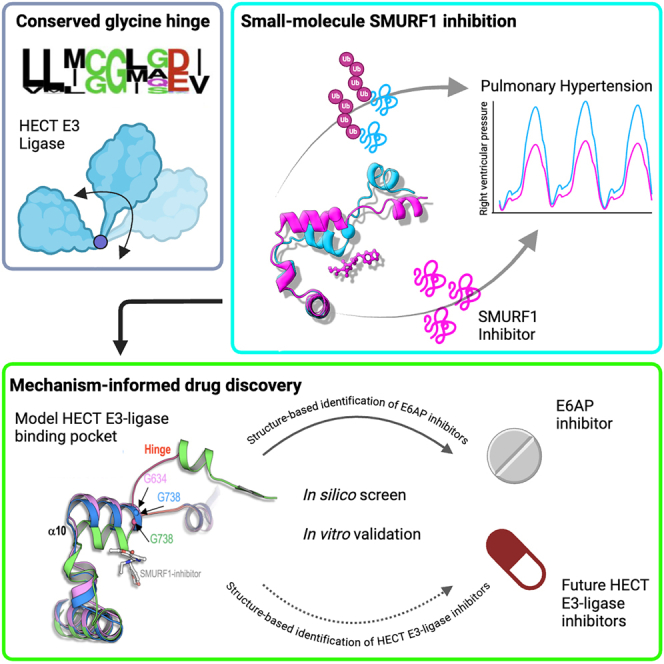

## Teaser

A large, unbiased biochemical screen discovers inhibitors of SMURF1 that reverse established pulmonary arterial hypertension by extending an α helix over a conserved glycine hinge to restrict the essential catalytic motion. Applying this mechanistic understanding to an *in silico* machine-learning-based screen identified inhibitors of the prototypic HECT E6AP, opening a new druggable space.

## Introduction

**Figure 1:**
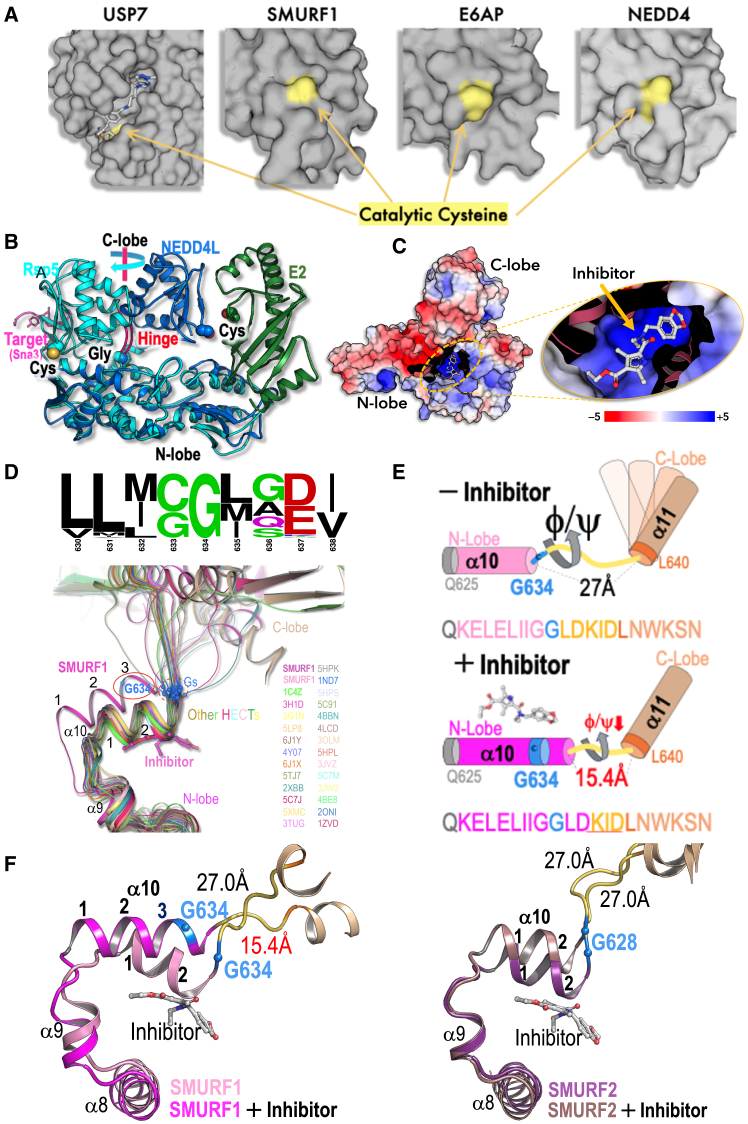
SMURF1 and SMURF2 HECT domain:inhibitor complex (A) The catalytic cysteine of HECT E3 ligases SMURF1, E6AP, and neural precursor cell expressed developmentally down-regulated protein 4 (NEDD4) is located on the external surface of the protein, in contrast to the active-site pocket location in the ubiquitin-specific-processing protease 7 (ubiquitin specific peptidase 7 [USP7]). (B) Superposition of HECT structures in two extreme rotation angles of the C-lobe. NEDD4L (blue), the catalytic cysteine is in close proximity to the E2 and ubiquitin (not shown); Rsp5 (cyan), the catalytic cysteine is facing the target (Sna3; magenta) and the C terminus of ubiquitin (not shown); and movement around the hinge is shown in Video S1. (C) Structure of inhibitor-bound SMURF1 (Cpd-8). A slice in the N-lobe reveals the cryptic cavity. Electrostatic surface potential was calculated with Adaptive Poisson-Boltzmann Solver with the indicated ± KT/e. (D) Logo sequence shows the residue conservation demonstrating the invariant G634 (Table S1). Superposition of 28 HECT structures, including inhibitor-bound SMURF1. The conserved glycine (blue spheres) are aligned at the stem of the hinge. The α helix10 (αH10) of SMURF1 (magenta) is elongated over the conserved glycine (G634) that is relocated within the αH10. (E) Schematic of the structural changes of αH10 and the altered length/flexibility of the hinge due to inhibitor binding. (F) Structural comparison of SMURF1 and SMURF2 with and without the inhibitor. See also Figures S1 and S2.

Ubiquitylation tightly regulates protein levels, activity, and localization in response to physiological stimuli. Dysregulation of this process is a key factor in the development of human diseases.^1^^,^^2^^,^^3^ The human genome encodes ∼650 E3 ligases, each ubiquitylating a limited number of targets, thereby providing specificity within the system. Unlike E1 ubiquitin-activating enzymes and deubiquitylases (DUBs), which present confined active-site pockets for inhibition,^4^^,^^5^^,^^6^ the active sites of ubiquitin E2-conjugating enzymes and E3 ligases protrude from the protein surface. This absence of an active-site pocket limits small-molecule binding and impedes computational approaches to identifying inhibitors (Figure 1A).

Artificial intelligence (AI), machine learning (ML), and other *in silico* methods have significantly enhanced our ability to identify and design novel small-molecule protein modulators. These algorithms rely on available protein structures. However, protein structures are dynamic, and current methodologies capture only the most stable conformations, overlooking transient pockets and cavities that could be exploited for drug design. These limitations have hindered the development of specific E3-ligase inhibitors.^6^^,^^7^^,^^8^

The Nedd4 subfamily, of the homologous to E6AP C terminus domain (HECT) group of E3 ligases, has direct links to human disease.^9^ Despite their therapeutic potential, there are currently no approved selective HECT inhibitors, making them an attractive starting point for the discovery of novel druggable features. Among these, the HECT family member SMURF1, first described in 1999,^10^ is implicated in multiple diseases for which new therapies are urgently needed, including cancer,^11^^,^^12^ bone,^13^^,^^14^ pulmonary,^15^^,^^16^ and central nervous system disorders.^17^ At the cellular level, SMURF1 acts as a negative regulator of bone morphogenetic protein (BMP) signaling, which modulates cell migration, proliferation, and apoptosis. The structure of SMURF1 is typical of a NEDD4 subfamily member, with a N-terminal C2 domain for cellular membrane localization, WW domains for target recognition, and a catalytic HECT domain that binds E2 and mediates target ubiquitylation through a surface-protruding catalytic cysteine.^9^^,^^18^^,^^19^^,^^20^ Ubiquitin transfer requires a motion facilitated by a flexible hinge that connects the HECT domain N- and C-lobes (Figure 1B; Video S1). Despite extensive research, little is known about the features that facilitate or inhibit this crucial motion.

Video S1. Structural rearrangements of SMURF1, related to Figure 1 A motion of the N- and C-lobes of SMURF1 around the flexible hinge is required to transfer ubiquitin from the catalytic cystine of the E2 to the protein target.

There is a compelling rationale for targeting SMURF1 in the treatment of pulmonary arterial hypertension (PAH). Heterozygous mutations of BMP receptor 2 (BMPR2)^21^^,^^22^ are present in 15%–40% of idiopathic disease,^23^ and reduced BMPR2 protein expression is observed in nongenetic PAH^24^ and animal models.^25^ Most BMPR2 mutations cause haploinsufficiency and reduced signaling. This reduction drives the proliferation and apoptosis resistance of pulmonary artery endothelial and smooth muscle cells,^26^^,^^27^ which contribute to increased pulmonary vascular resistance, right heart failure, and death.^28^ Data demonstrate that enhancing BMP signaling provides therapeutic benefits in experimental PAH^29^^,^^30^ and that restoring balance of the BMP/transforming growth factor beta (TGF-β) pathways provides clinical benefit.^31^^,^^32^

We have previously reported that SMURF1 expression is increased in patients with PAH and that global deletion of SMURF1 provides allele-dependent protection against experimental PAH.^16^ Considering the established Mendelian association of mutations in the BMP pathway with the disease, the central role of BMP/TGF-β signaling in PAH development,^21^^,^^33^^,^^34^^,^^35^^,^^36^ and the negative regulatory role of SMURF1 in BMP signaling,^10^^,^^16^ we hypothesized that inhibition of SMURF1 could augment BMP signaling and thereby offer a potential treatment for PAH.

To circumvent the limitations of *in silico* methods, we performed a large, unbiased screen of 1.1 million compounds and identified potential SMURF1 inhibitors. Subsequent X-ray crystallography studies revealed compounds that bind a cryptic cavity distant from the SMURF1 catalytic cysteine.

Here, we describe at atomic resolution an allosteric inhibitor mechanism that restores BMP signaling and reverses established disease in experimental model animals. We further leverage the deep understanding of the allosteric mechanism to discover *in silico* inhibitors for other HECT E3s.

## Results

### Selective inhibitors restrict crucial glycine-hinge motion within the HECT domain

To overcome the lack of a known active-site pocket, we developed a time-resolved fluorescence resonance energy transfer (TR-FRET)-based assay reporting SMURF1 self-ubiquitylation (Figure S2A). Using this system, we undertook a large, unbiased high-throughput screen (HTS) of 1.1 million compounds. Primary hits were further screened using biochemical selectivity and cell-based assays designed to prioritize and optimize molecules for specific SMURF1 inhibition (Figures S2B and S2C). This process revealed three-chemical series with favorable drug-like properties: piperidine sulfonamides, pyrazolones, and pyrroles (Figures S2D and S2E).^37^ Although SMURF1 and SMURF2 share a high degree of protein sequence homology (86% identity), we identified potent inhibitors that were selective for SMURF1 over SMURF2 (Figures S2D and S2E), which were structurally distinct from those reported in prior studies.^38^^,^^39^^,^^40^

To gain structural insights into the mechanism of selective inhibition, we crystallized the HECT domains of both SMURF1 and SMURF2, without and with a representative SMURF1 inhibitor, compound-8 (Cpd-8), and determined structures at 2.05–2.75 Å resolution (Figures 1C, 1D, and S2A). In the absence of the inhibitor, the HECT domains of SMURF1 and SMURF2 adopted a highly similar architecture, comprising N- and C-lobes connected by a flexible glycine-containing hinge (residues G634–D639) (Figures 1B–1D; Video S1). The superimposed HECT domain structures of all family members suggest that the motion between the N- and C-lobes around this hinge is important for ubiquitin transfer from E2 to the target protein via the HECT C-lobe.^41^ The range of this motion is defined by the conformation adopted at two extremes: the ubiquitin∼E2∼HECT complex and the ubiquitin∼HECT∼target complex.^42^^,^^43^^,^^44^ Function of the hinge is facilitated by its length and the flexibility provided by amino acid residues with a tolerance to a wide range of ϕ/Ψ dihedral angles (Figures 1D and 1E).

Highly conserved amino acid sequences are often critical to protein structure and function. To determine amino acid sequences critical for SMURF1 activity, we employed a fast Fourier transformation-based multiple alignment. These analyses demonstrated that G634, which stems the SMURF1 hinge, is invariant in all HECT sequences across animal, plant, and fungal kingdoms and in all 28 HECT domain structures (Figure 1D; Table S1). The functional importance of this invariant glycine is reinforced by the UBE3A/E6AP G738E mutation, which causes Angelman syndrome.^45^

Across all 28 HECT domain structures (including SMURF1), the α helix10 (αH10) comprises two and a half turns and ends with the conserved glycine, which provides flexibility between the N- and C-lobes through its high tolerance of ϕ/Ψ dihedral angles (Figure 1D). In complex with SMURF1, the inhibitor is buried in an N-lobe cryptic cavity, with only 2% of its surface exposed (Figures S2B–S2D). Compared with unbound SMURF1, the αH10 of the SMURF1-inhibitor complex is elongated by one and a half turns over the conserved glycine (G634). This shortens the hinge (from 27.0 to 15.4 Å) and replaces G634 at the stem of the flexible hinge with lysine (K637), an amino acid with a lower tolerance of the ϕ/Ψ dihedral angles (Figures 1D and 1E). These crystallographic data were confirmed by unbiased refinement using electron density with Sigma-A *mFo-DFc* simulated-annealing omit maps (Figures 1F, S2A, and S2E).^46^

**Figure S1:**
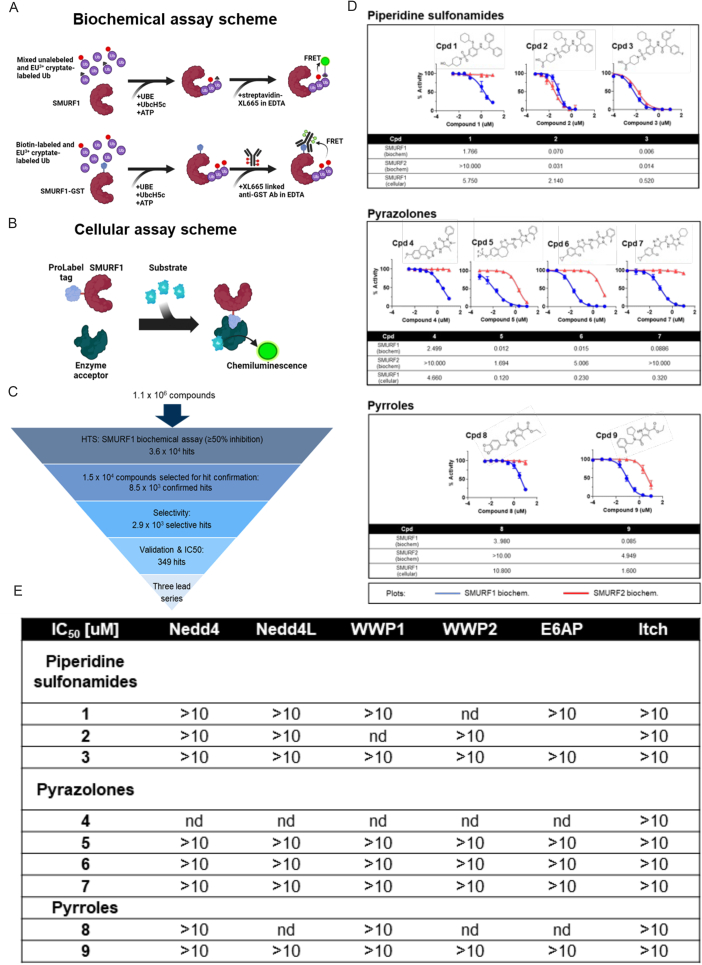
HTS strategy and activity of lead compounds from prioritized chemical series, related to Figure 1 (A) Schematic showing the biochemical TR-FRET assays used for detection of SMURF1 self-ubiquitylation. (B) Cell-based assay: detection of E3 ligase self-ubiquitylation, assay used for the validation screen and selectivity profiling. (C) Process used for triaging of initial hits through to identification of lead series. (D) Overview of pharmacological activity for three prioritized chemical hit classes. Plots show dose-response in SMURF1 (blue) and SMURF2 (red) biochemical assays (*n* = 4–11). The tables below indicate half-maximal inhibitory concentration (IC50) values for respective inhibitors in SMURF1 and SMURF2 biochemical assays and SMURF1 cellular assay (*n* = 2–474). (E) Selectivity of SMURF1 inhibitors across representative structurally related HECT family members (*n* = 4–20).

The structure of SMURF2 in complex with Cpd-8 demonstrated binding of the same cryptic cavity as the SMURF1-inhibitor complex. However, in contrast to SMURF1, inhibitor binding did not elongate the SMURF2 αH10, and the invariant glycine (G628) stems the hinge in both the inhibitor-bound and -unbound forms (Figure 1F). Consistent with the absence of allosteric structural changes, SMURF2 activity was not reduced by inhibitor binding (Figure S1D).

Based on these structures, we hypothesized that SMURF1 activity is inhibited due to a reduction in the length and flexibility of the glycine hinge, induced by the allosteric activity of the inhibitor.

### SMURF1 escape mutants resist allosteric inhibition

**Figure 2:**
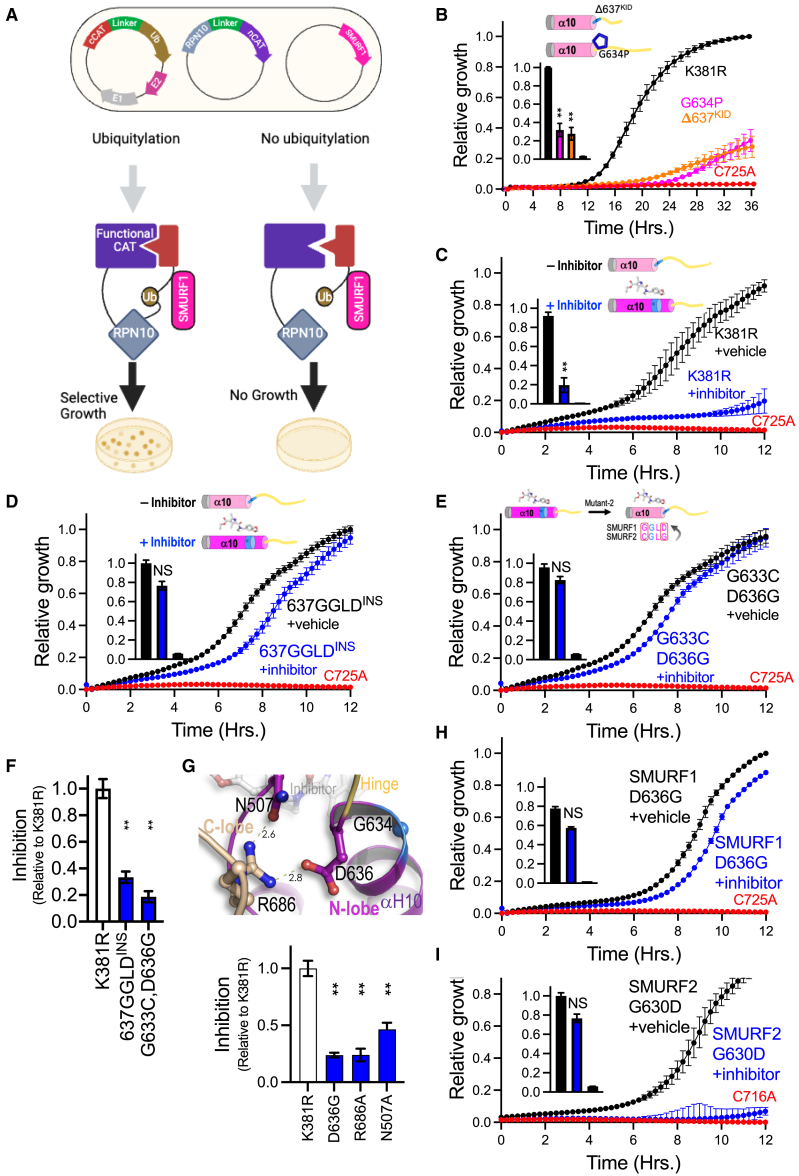
Allosteric inhibition of SMURF1 (A) Illustration of the *E. coli* split-CAT system showing target ubiquitylation resulting in CAT assembly, resistance, and selective growth. (B) Replacement of the SMURF1 conserved glycine with proline (G634P), an amino acid with a covalently linked side chain that limits hinge flexibility and a deletion that reduced hinge length (Δ637^KID^), both reduce ubiquitylation. (C) Cpd-8 (blue) reduces ubiquitylation compared with vehicle (black). (D) SMURF1 escape mutant-1 (insertion of GGLD downstream to D636) shows resistance activity to the inhibitor. (E) SMURF1 escape mutant-2 (replacing indicated residues with SMURF2 residues) shows significant resistance to the inhibitor. (F) Relative inhibition of the escape mutants. (G) SMURF1 G636 forms a non-covalent bond that stabilizes the elongated αH10. Mutation of each of the three residues (D636G, R686A, and N507A) results in escape from inhibition. (H) Escape mutant-3 (D636G mutation replaces the amino acid that stabilizes the elongated αH10 with one that does not form a non-covalent bond) shows significant resistance to the inhibitor. (I) Susceptibility mutant (G630D, replacing indicated residues of SMURF2 with SMURF1; SMURF1lation) shows the sensitivity of mutant SMURF2 to inhibitor. All data: *n* = 4 replicates; mean ± SD, ^∗∗^*p* < 0.01, ^∗∗∗^*p* < 0.001 Student’s t test or one-way ANOVA with Dunnett’s correction as appropriate. See also Figures S2 and S3.

**Figure S2:**
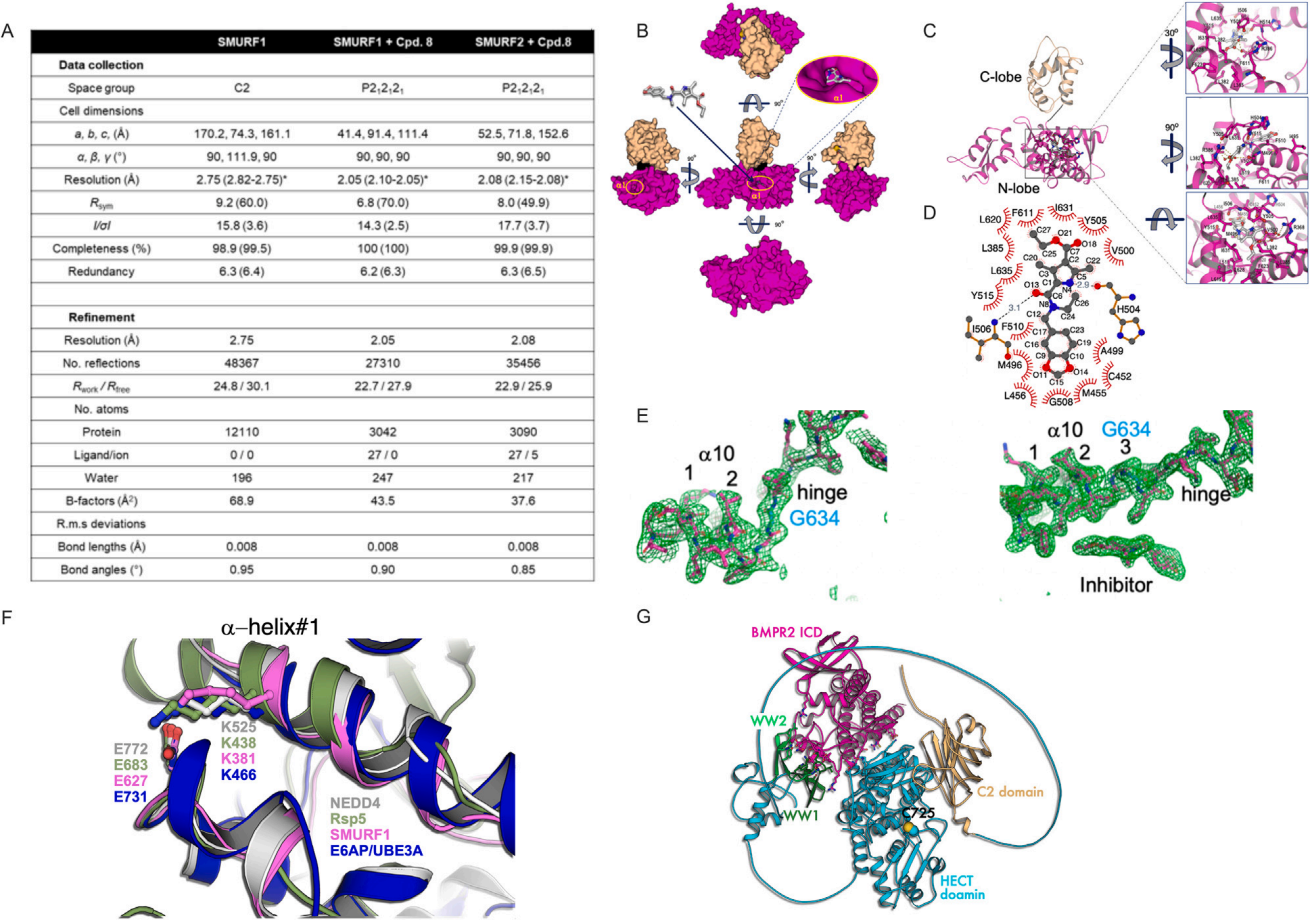
Structural details of inhibitor binding to SMURF1, conservation of lysine residues on α helix #1, and AlphaFold model of the SMURF1:BMPR2 complex, related to Figures 1, 2, 4, and 6 (A) Crystallography parameters. ^∗^The highest resolution shell is shown in parenthesis. (B) Surface representation of SMURF1 shows that the inhibitor is minimally exposed. (C) Detailed structural insight into the SMURF1—inhibitor complex, with a zoomed view of the binding cavity from three different angles. (D) 2D representation of the inhibitor binding site, rendered using LigPlot+ v.2.2 https://www.ebi.ac.uk/thornton-srv/software/LigPlus/. (E) mFo-DFc simulated-annealing electron density omit map showing the α10 and the hinge region without (left) and with (right) the inhibitor. The map was calculated by omitting the entire model of the α 10, the hinge, and the inhibitor using simulated-annealing sigma A analysis contoured at 2.75 (SMURF1, apo) and 2.05 Å (SMURF1, Cpd-8) at 3σ. (F) Structures of HECT ligase showing conservation of lysine residues on α helix #1. Superimposing the structures of Rsp5 (3OLM), NEDD4 (2XBB), and SMURF1 and AlphaFold model of E6AP/UBE3A HECT domains shows the conservation of lysine (K) residues on α helix #1, previously demonstrated to undergo self-ubiquitylation that downregulates the ligase activity.^52^ (G) AlphaFold model of the SMURF1:BMPR2 complex. The sequences of full-length SMURF1 and the intracellular domain of BMPR2 were modeled in AlphaFold3. The domains of SMURF1 are indicated. Residues predicted to participate in binding are shown as ball-and-sticks. The model suggests that WW1 and WW2 domains directly interact with the kinase domain of BMPR2.

**Figure S3:**
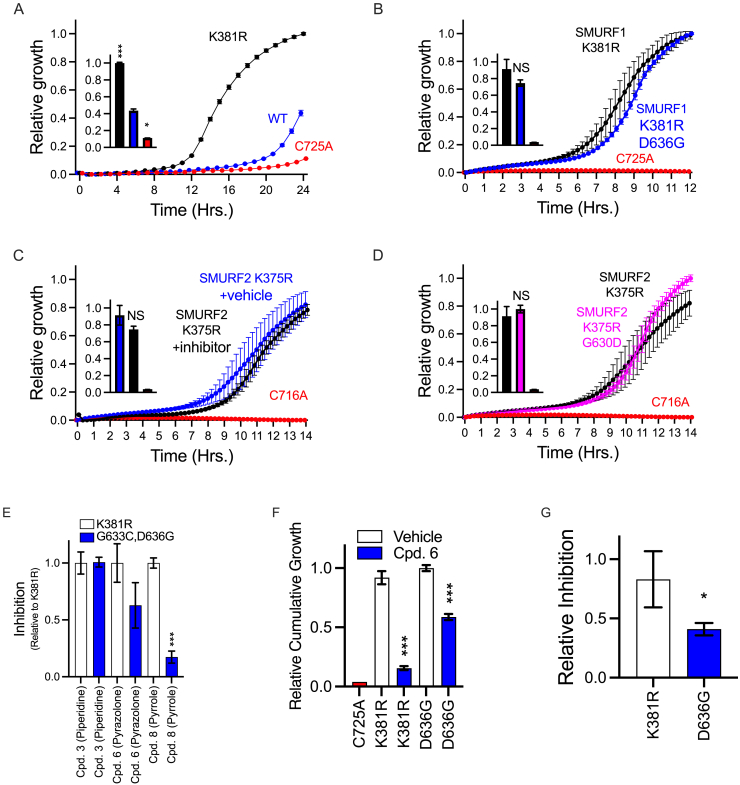
Split-CAT-based *E. coli*, related to Figures 2, 4, and 6 System monitoring SMURF1 and SMURF2 activity and evaluation of structure-based allosteric mechanism of serries representative compounds. (A) SMURF1-dependent ubiquitylation of Rpn10: wild-type (blue), K381R mutant (black), and catalytically inactive C725A (red). (B) SMURF1-dependent ubiquitylation of the phosphomimetic peptide of SMAD1 (black) with D636G mutation (blue). (C) SMURF2-dependent ubiquitylation of Rpn10 with (black) or without (blue) Cpd-8. (D) SMURF2-dependent ubiquitylation of Rpn10 (black) with G630D mutation (pink). Growth curves with inset bars representing area under the curve (AUC), *n* = 4 replicates, mean ± SD. The SMURF1 inhibition modes of representative compound from each of the three-chemical series (piperidine, pyrazolone, and pyrrole): Cpd-3, Cpd-6, and Cpd-8 were assessed in the split-CAT assay. The compounds were assessed against hyperactive (K381R mutant)—white bars or an escape mutant (K381R, G633C, and D636F)—blue bars. (E) Shows relative inhibition of SMURF1 by each of the compounds using Rpn10 as ubiquitylation target reporter (mean ± SD; *n* = 3 replicates). (F and G) As in (A), but Rpn10 was replaced with phosphomimetic peptide of SMAD1 as ubiquitylation target reporter. *n* = 4 replicates, mean ± SD.

To biochemically test the structure-based hypothesis of the SMURF1 glycine-hinge function, we constructed mutants that either restrict the ϕ/Ψ dihedral angles or shorten the hinge length. We employed an *E. coli* split chloramphenicol acetyltransferase (split-CAT) system as a reporter to measure SMURF1 activity using Rpn10, a protein that is efficiently ubiquitylated by most E3 ligases *in vitro*, as a target (Figures 2A, S2F, and S3).^47^^,^^48^^,^^49^^,^^50^ In this assay, ubiquitylation results in the functional assembly of the split-CAT, allowing for selective bacterial growth, which serves as a quantitative reporter of ubiquitylation efficiency (Figure 2A). To maximize the assay amplitude, we constructed a hyperactive ΔC2-K381R SMURF1 mutant that lacks the self-ubiquitylation-dependent regulation previously reported for NEDD4/Rsp5 (Figures S2F and S3A).^51^^,^^52^^,^^53^ When expressed in the split-CAT-based *E. coli* reporter system, the hyperactive ΔC2-K381R SMURF1 mutant increased Rpn10 ubiquitylation compared with both the wild-type SMURF1 and the catalytically inactive C725A SMURF1 (Figure S3A; ΔC2-K381R SMURF1 and ΔC2-K375R SMURF2 are used as the basis for all further mutation and inhibition assays). To investigate the importance of hinge flexibility and length for SMURF1 function, two classes of mutants were constructed: (1) a mutant with reduced hinge flexibility, created by substituting the conserved glycine with proline (G634P), which reduces ϕ/Ψ angles due to a covalently linked side chain; and (2) a mutant with reduced hinge length, created by deleting three amino acid residues (Δ637^KID^). Both mutants reduced ubiquitylation activity, confirming the critical role of glycine-hinge flexibility and length (Figure 2B).

Further tests showed that Cpd-8 significantly attenuated the activity of SMURF1 (Figure 2C). We therefore engineered two SMURF1 mutants designed to resist the effect of the allosteric inhibitor: (1) a mutant that restores the flexibility and length of the hinge (637GGLD^INS^), constructed by inserting a GGLD sequence immediately downstream of the elongated αH10 induced by inhibitor binding (Figure 2D); and (2) a mutant that maintains inhibitor binding but resists allosteric αH10 elongation, constructed by replacing two critical residues at the C-terminal end of αH10 with those present in SMURF2 (G633C, D636G, named SMURF2lation; Figure 2E). Neither 637GGLD^INS^ nor the G633C, D636G double mutant significantly altered SMURF1 activity in the absence of the inhibitor. Notably, both mutants retained more than 75% activity in the presence of Cpd-8, indicating that preservation of the glycine-hinge flexibility and length provides effective escape from the inhibitor (Figures 2D–2F).

### A lock mechanism stabilizing elongated αH10

Further structural analyses suggested that a network of non-covalent bonds between D636, R686, and N507 stabilizes the allosterically elongated αH10 of SMURF1, induced by compound binding (Figure 2G). Based on this observation, we modeled the transition of the αH10 and glycine hinge from the unbound to the compound-bond state (Video S2). The analysis showed that in unbound-SMURF1, I631 of the αH10, along with other nearby residues, occupies the cryptic cavity, preventing small-molecule inhibitor binding. In the presence of Cpd-8, the αH10 is displaced, inducing allosteric elongation. In this altered confirmation, an electrostatic bond is formed between the αH10 D636 and C-lobe R686. R686 is further stabilized by N507, which is allosterically shifted toward R686. To assess the importance of this network of non-covalent bonds, SMURF1 mutants predicted to disrupt it were generated, and enzymatic function was evaluated. SMAD family member 1 (SMAD1), a known target of SMURF1, exhibits enhanced binding affinity and specificity upon phosphorylation.^54^^,^^55^ Using the split-CAT system, we expressed a phosphomimetic-SMAD1 (S210,214E) to further optimize binding and specificity. The independent mutations D636G, R686A, and N507A did not alter enzymatic activity but conferred resistance to inhibition by Cpd-8, thereby demonstrating the importance of αH10 stabilization of the inhibited form of SMURF1 (Figures 2G, 2H, and S3B).

Video S2. The effect of allosteric inhibition on the structural rearrangements of SMURF1, related to Figure 2 (A) In unbound-SMURF1, I631 of the αH10 (and other nearby residues) occupies the cryptic cavity, which is therefore not available for small molecule inhibitor binding.
(B) In the presence of Cpd-8, the αH10 is pushed away, resulting in allosteric elongation. In this altered confirmation, an electrostatic bond is formed between D636 in the αH10 and R686 located on the C-lobe.

SMURF1 R686 is conserved in SMURF2 as R680, but SMURF1 D636 is replaced with G630 in SMURF2, meaning that the network of non-covalent bonds stabilizing the elongated αH10 in SMURF1 cannot be formed in SMURF2. Consistent with this, SMURF2 was not inhibited by Cpd-8 (Figure S3C). To examine whether introducing the residues required for the formation of the stabilizing bond network would render SMURF2 sensitive to inhibition, we engineered SMURF2 with a G630D substitution (named SMURF1lation). Activity of the G630D SMURF2 against the phosphomimetic SMAD1 peptide target was not significantly altered (Figure S3D). However, the G630D SMURF2 was susceptible to inhibition by Cpd-8 (Figure 2I).

Together, these data confirm that binding of the inhibitor to SMURF1 induces elongation of the αH10 over a conserved glycine hinge, stabilized by a network of non-covalent bonds, which reduces flexibility and length of the glycine hinge that is essential for catalytic action. The resistance of SMURF2 to inhibition is likely conferred by G630, which, in the presence of compound, does not form the bond network required to lock the elongated αH10.

### A mechanism applicable to pyrazolone and pyrrole compounds

The allosteric mechanism described was proposed based on the crystal structures of SMURF1 and SMURF2 with and without Cpd-8 and validated in the split-CAT system with the same compound. We next sought to investigate whether compounds from each of the three-chemical series identified in the unbiased screen function through the same allosteric mechanism. Using the split-CAT system, we found that Cpd-3 (piperidine), Cpd-6 (pyrazolones), and Cpd-8 (pyrrole) all inhibited ubiquitylation, corroborating the findings from the TR-FRET assay. However, in SMURF1 mutants engineered to increase the flexibility and length of the glycine hinge, only Cpd-6 and Cpd-8 showed reduced inhibition. This confirmed that Cpd-6 and Cpd-8 function through the identified allosteric mechanism, while Cpd-3 functions via an alternate mechanism (Figure S3E).

To gain structural insights into the mechanism of inhibition, we sort to crystallize SMURF1 and SMURF2 with piperidine compounds. Cpd-3 did not yield co-crystals with either SMRUF1 or SMURF2. However, crystals of the HECT domain of SMURF2 without and with Cpd-2 were obtained, and structures were determined at 2.05–2.75 Å resolution. Cpd-2 bound the same cryptic pocket between the C- and N-lobe of SMURF2 as Cpd-8. However, in contrast to Cpd-8, Cpd-2 extended from the pocket to physically interact with the C-lobe of SMURF2, preventing the required motion of the glycine hinge without allosteric elongation αH10. Due to its drug-like properties, we further evaluated the mechanism of inhibition of Cpd-6 using SMAD1, a native SMURF1 target. By demonstrating that escape mutants resisted inhibition, we confirmed that Cpd-6 functions through the same allosteric mechanism as Cpd-8 (Figures S3F and S3G). This finding is consistent with the specificity of the compounds in that Cpd-3 inhibits both SMURF1 and SMURF2, whereas Cpd-6 and Cpd-8 inhibit only SMURF1 (Figure S1D).

### SMURF1 is overexpressed in PAH

**Figure 3:**
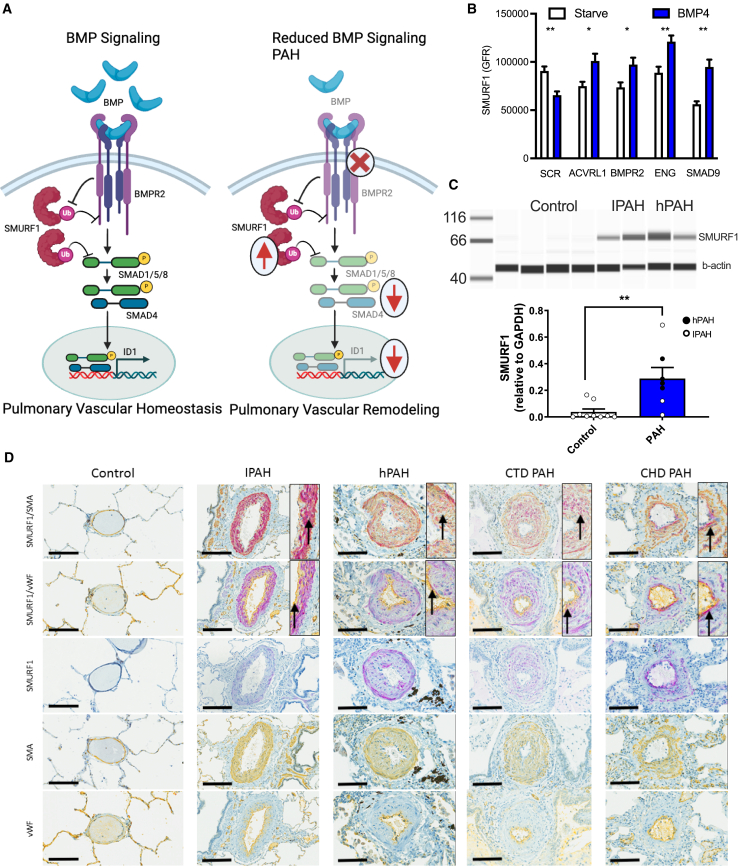
SMURF1 expression in PAH (A) Schematic representation of canonical BMP signaling resulting in SMAD1/5/8 phosphorylation, in nuclear translocation of SMAD4 and ID1 expression, and its negative regulation by SMURF1-mediated ubiquitylation and degradation of BMPR2 and SMAD1/5/8. (B) In HEK293 cells stably transfected with GFP-tagged SMURF1, BMP4 stimulation results in a decrease in GFP signal and short interfering RNA (siRNA) knockdown of ACVRL1, BMPR2, Endoglin (ENG), or SMAD9, and BMP4 stimulation results in increased GFP signal. *n* = 3 separate experiments; presented as mean ± SEM. ^∗^*p* < 0.05, ^∗∗^*p* < 0.01, relative to untreated, unpaired Student's t test. (C) Expression of SMURF1 is increased in PASMC from patients with idiopathic and heritable PAH when compared with PASMC from patients without PAH. *n* = 6 PAH donor lines and *n* = 9 control lines; presented as mean ± SEM. ^∗∗^*p* < 0.01, unpaired Student's t test. (D) SMURF1 expression in the pulmonary artery intima and media in patients with PAH. SMURF1 (purple) protein co-localization with an endothelial (von Willebrand factor [vWF], yellow) or smooth muscle marker (Alpha Smooth Muscle Actin [αSMA], yellow) is indicated by a red/brown color shift. Representative images were obtained from 19 controls and 33 patients with PAH. Scale bar, 60 μm. Arrows indicate areas of co-localization. See also Figure S4.

**Figure S4:**
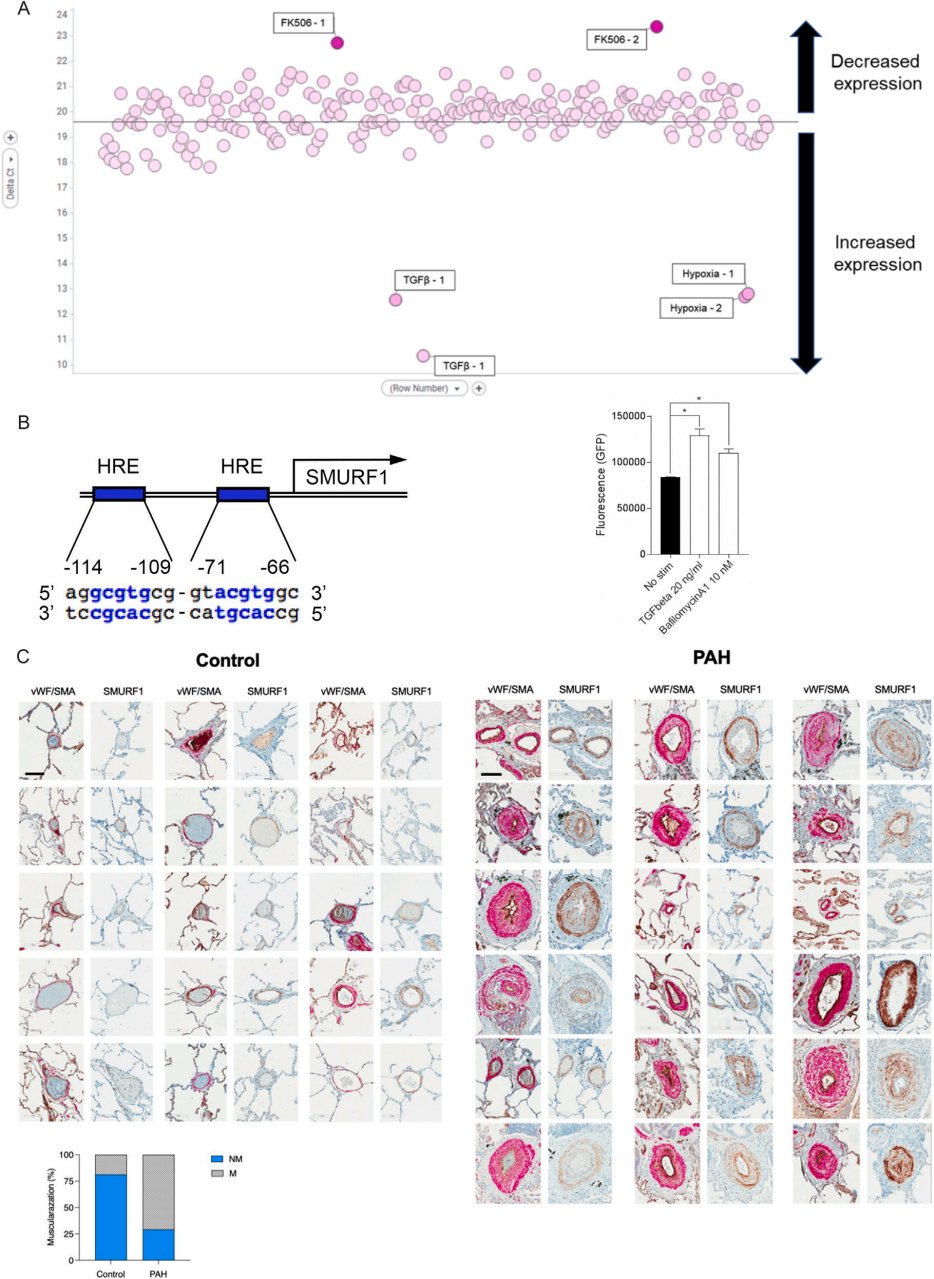
SMURF1 expression in patient samples, related to Figure 3 (A) Screen for modulators of SMURF1 mRNA abundance in PASMCs. Human PASMCs from PAH patients were treated with agents that are associated with PAH, including cytokines, chemokines, growth factors, and small-molecule pathway probes, and were cultured under hypoxic conditions to identify modulators of SMURF1 mRNA (*n* = 2 donors). The figure shows normalized Ct values (ΔΔCt method). The BMP agonist FK506 (tacrolimus) was found to reduce SMURF1 expression. Both hypoxic conditions and TGF-β1 treatment increase SMURF1 expression, supporting SMURF1 induction as part of a feedback loop regulating BMP and TGF-β1 signaling. (B) *In silico* analysis indicates that the *SMURF1* promoter contains two bindings sites for the hypoxia-regulated transcription factor, hypoxia-inducible factor 1 subunit alpha (HIF1α). The human SMURF1 promoter was stably transfected into HEK293 cells upstream of a Green Fluorescence Protein (GFP) encoding sequence. Administration of TGF-β1 and bafilomycin A1 increased GFP signal. (C) Representative histology sections of explanted lungs from patients with and without pulmonary arterial hypertension. Dual staining was performed for vWF (brown) and αSMA (pink), and single staining for SMURF1 (brown). Each set of two images represents a unique patient (total: *n* = 15 control and *n* = 18 PAH patients). Scale bar, 60 μm. Plot: quantification of pulmonary vascular muscularization in above histological images (M, full muscularization; NM, no muscularization).

Human genetic studies implicate reduced BMP signaling as an initiating factor in the onset of PAH.^21^^,^^22^^,^^33^^,^^34^^,^^35^^,^^36^ Canonical BMP signaling is initiated by ligand binding of the BMP receptor complex, resulting in phosphorylation of SMAD1/5/8, nuclear translocation of SMAD4, and increased inhibitor of DNA binding 1 (ID1) expression (Figure 3A).^56^ Simultaneously, upon stimulation, tribbles pseudokinase 3 (TRIB3) is released from the BMP receptor complex, mediating degradation of SMURF1 (reducing levels of SMURF1), stabilization of SMAD1/5/8, and potentiation of the pathway.^10^ As such, BMP signaling increases, and reduced BMP signaling reduces the availability of downstream signaling mediators through SMURF1. SMURF1 expression is also increased by hypoxia or TGF-β signaling, both of which are central drivers of vascular remodeling (Figures S4A and S4B), further implicating SMURF1 as a key mediator of reduced BMP signaling and the subsequent vascular remodeling observed in PAH.

To understand the effect of disease-associated mutations on SMURF1 regulation, we engineered HEK293 cells to stably express green fluorescent protein (GFP)-tagged SMURF1. Consistent with prior reports, BMP4 stimulation reduced the GFP-tagged SMURF1 levels (Figure 3B).^56^ However, following small interfering RNA (siRNA)-mediated reduction of activin A receptor-like type 1 (ACVRL1), BMPR2, and endoglin (ENG), the level of GFP-tagged SMURF1 increased in response to BMP4 stimulation (Figure 3B).

PAH is characterized by remodeling of endothelial and smooth muscle cells of the small pulmonary arteries,^26^^,^^27^ in which increased SMURF1 expression has been described.^57^^,^^58^ To investigate the penetrance of this finding, we examined SMURF1 expression in small pulmonary arteries in 33 PAH patients. We observed significantly increased SMURF1 expression in endothelial and smooth muscle cells in all samples from PAH patients compared with 19 patients without PAH. To our knowledge, this is the first report of SMURF1 expression in PAH-affected lungs at the cellular level, using the largest sample size to date. Thus, this study represents the most comprehensive assessment of SMURF1 expression in the lungs of PAH patients (Figures 3C, 3D, and S4C).

### SMURF1 directly ubiquitylates SMAD1 and BMPR2

**Figure 4:**
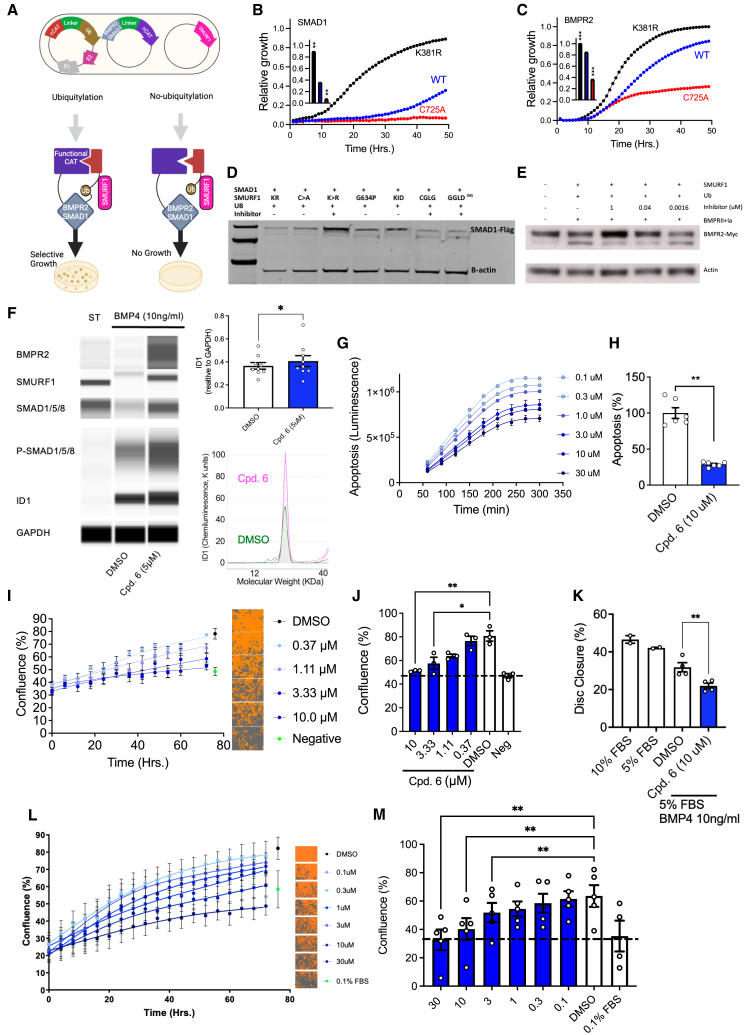
SMURF1 inhibitors restore BMP signaling and pulmonary vascular cell homeostasis (A) Scheme of SMURF1-BMPR2 and SMAD1 split-CAT based *E. coli* selection system, showing target ubiquitylation resulting in CAT assembly, resistance, and selective growth. (B and C) Hyperactive K381R (black) increases and catalytically inactive C725A (red) reduces SMURF1-dependent direct target ubiquitylation of SMAD1 (B) and BMPR2 (C) (inset represents area-under-the-curve of relative growth; *n* = 3, ^∗∗^*p* < 0.01, ^∗∗∗^*p* < 0.001, one-way ANOVA with Dunnett’s correction). (D) Representative western blot demonstrating stabilization of overexpressed SMAD1 in HEK cells in the presence of SMURF1 inhibitor. Mutations that reduce the flexibility (G634P) and length (Δ637^KID^) of the hinge result in reduce SMURF1 activity. Mutations that preserve the flexibility (G633C, D636G [GGLD]) and length (637GGLD^INS^) of the SMURF1 glycine hinge escape the effect of the inhibitor. (E) Representative western blot demonstrating stabilization of overexpressed BMPR2-myc in HEK cells in the presence of SMURF1 inhibitor. (F) Immunoblotting of BMPR2, SMURF1, SMAD1/5/8, phosphorylated SMAD1/5/8, ID1, and glyceraldehyde-3-phosphate dehydrogenase (GAPDH) in PASMC cultured without or with BMP4 and SMURF1 inhibitor (Cpd-6) (*n* = 9 separate experiments across cells from 3 PAH donor lungs, mean ± SEM). (G and H) Quantification of apoptosis in PAECs. (G) Representative time curve (*n* = 5 technical replicates, mean ± SD) and (H) group data at 300 min with Cpd-6 or vehicle (*n* = 6 separate donors, mean ± SEM). (I and J) Quantification of proliferation: (I) representative time course (*n* = 5 technical replicates, mean ± SD) in PAEC with cell confluence mask for each dose of Cpd-6 or vehicle (orange) and (J) group data (*n* = 3 separate PAEC donors; mean ± SEM). (K) Migration of PASMCs with Cpd-6 or vehicle measured via disc closure assay (*n* = 2–3 separate donors). (L) Representative time course plots showing proliferation of PASMC from an idiopathic PAH patient with Cpd-6 or vehicle with cell confluence mask for each dose (orange) (*n* = 5 technical replicates per concentration from one idiopathic PAH donor line, mean ± SD). (M) Group proliferation data at 72 h in PASMCs from patients with idiopathic or hereditary PAH (*n* = 4–5 separate donor lines, mean ± SEM). ^∗^*p* < 0.05, ^∗∗^*p* < 0.01, one-way ANOVA with Dunnett’s correction. See also Figures S2, S3, and S5.

BMPR2 and SMAD1 are key mediators of BMP signaling, both of which are reduced in patients (Figure 3A). SMURF1 has been reported to directly ubiquitylate SMAD1,^10^ but studies suggest that BMPR2 ubiquitylation is mediated through SMAD6 or SMAD7.^59^ SMAD1 and the intracellular domain of BMPR2 were expressed in the split-CAT-based *E. coli* selection system to assess if each were direct targets of SMURF1 (Figure 4A; Video S3). Compared with wild-type SMURF1, the hyperactive SMURF1 increased ubiquitylation of both targets, while the C725A catalytic inactive mutant reduced ubiquitylation (Figures 4B and 4C). These results are corroborated by the AlphaFold model of the SMURF1:BMPR2 interaction, suggesting that two WW domains but not the C2 domain of SMURF1 interact with BMPR2 (Figure S2G). As *E. coli* lacks the accessory proteins that bridge SMURF1 to BMPR2, these data demonstrated that BMPR2 is a direct target of SMURF1.

Video S3. SMURF1-BMPR2 split-CAT assay in *E. coli*, related to Figure 4 Time-lapse imaging shows BMPR2 ubiquitylation resulting in Split-CAT assembly and selective growth on chloramphenicol agar plates. SMURF1-dependent (direct) BMPR2 ubiquitylation promotes bacterial growth. Controls: the hyperactive SMURF1 K381R mutant increases growth, while the catalytically inactive SMURF1 C725A mutant reduces growth.

To evaluate the relevance of this finding to mammalian cells, SMAD1 and BMPR2 were overexpressed in HEK-293 cells with key components of the ubiquitylation cascade. Inhibition of SMURF1 increased levels of both SMAD1 and BMPR2 (Figures 4D and 4E). Consistent with the proposed allosteric mechanism of inhibition of SMURF1, mutations designed to reduce the length and flexibility of glycine hinge (G634P and Δ637^KID^) increased SMAD1 levels. Mutations designed to preserve hinge length and flexibility (G633C, D636G double mutant, and 637GGLD^INS^) enabled escape from inhibition (Figure 4D). Together, these data demonstrate allosteric inhibition of SMURF1 in mammalian cells and, through its direct targets BMPR2 and SMAD1, modulation of two critical points within the BMPR2 pathway.

### SMURF1 inhibition augments BMP signaling

**Figure S5:**
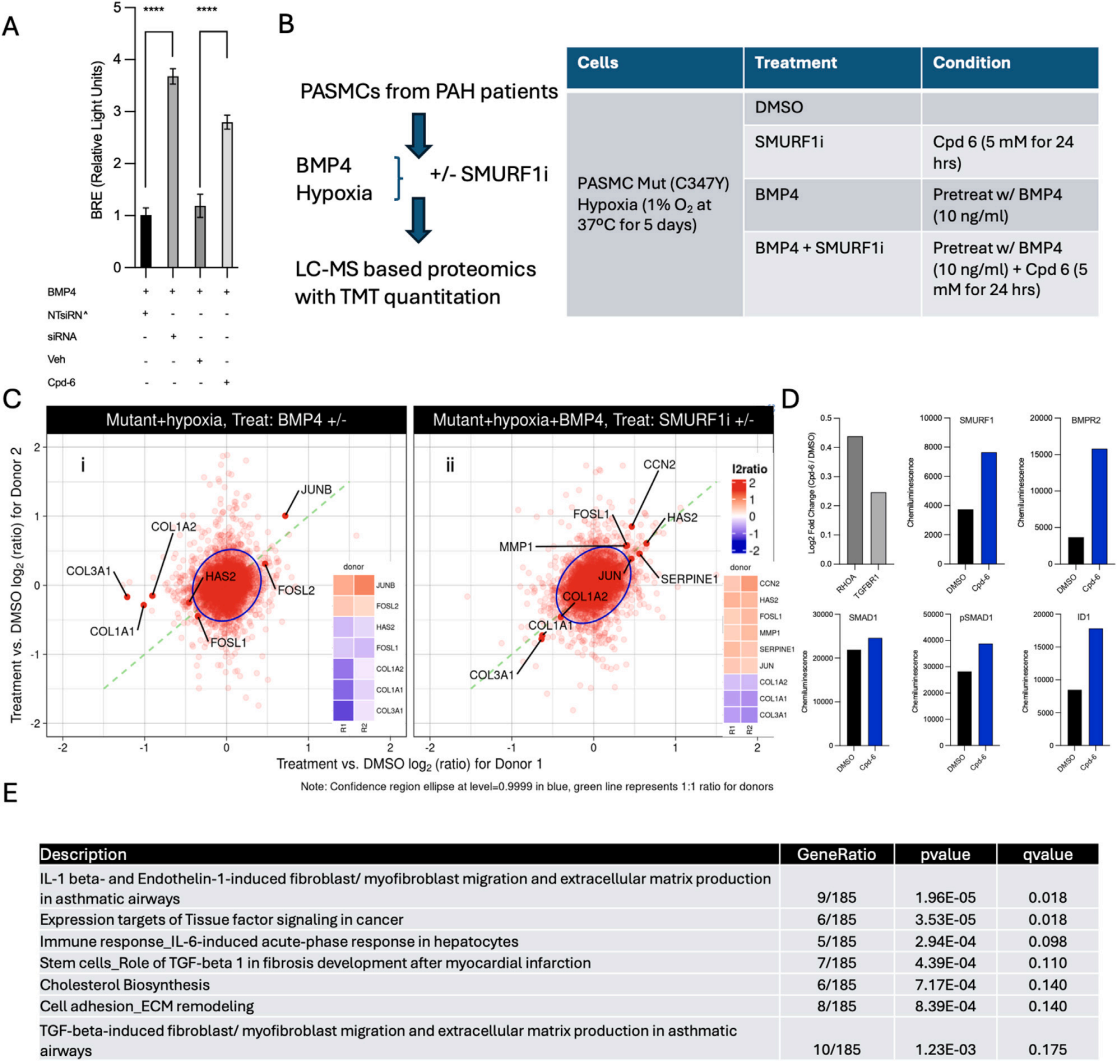
BMP signaling and proteomic effects of SMURF1 inhibition in mammalian cells, related to Figure 4 (A) Comparison of small-molecule SMURF1 inhibition and siRNA-mediated SMURF1 knockdown on BMP signaling (BMP response element – ID1 promoter activation) in HEK293 cells. (B–D) Summary of expression proteomics experiments comparing PASMCs treated under hypoxic conditions ±BMP4 or ±SMURF1 inhibitor Cpd-6 (SMURF1i). (B) Experimental workflow. Table: sample conditions for PASMC BMPR2 (C347Y) mutant cells undergoing TMT quantitative proteomics profiling at 24 h. (C) Log_2_ ratios of protein abundances for treated versus control (DMSO) in selected conditions. Data represent two biological replicates (*n* = 2) per treatment condition in a single experiment derived from individual donors. Highlighted proteins correspond to “IL-1 beta- and endothelin-1-induced fibroblast/myofibroblast migration and extracellular matrix production in asthmatic airways” gene set, which was significantly enriched among dysregulated proteins, only in condition (B) (ii). Inserts show heatmap for ratios for donors 1 and 2 for proteins highlighted in the plot. (D) Log_2_ ratio of known SMURF1 targets RhoA and TGFBR1 detected in proteomic study and chemiluminescence of SMURF1, BMPR2, SMAD1, pSMAD1, and ID1 in samples used for proteomic studies measured by western (not detected by proteomics). (E) Summary of significantly enriched terms for mutant cells under hypoxic conditions pre-treated with BMP4, followed by treatment with SMURF1 inhibitor or DMSO.

To investigate the role of allosteric SMURF1 inhibition on BMP signaling in disease-relevant cells, pulmonary artery smooth muscle cells (PASMCs) from patients with idiopathic and heritable PAH were treated with BMP4 and cultured in the presence of a SMURF1 inhibitor or vehicle. Compared with vehicle control, allosteric inhibition of SMURF1 led to increased levels of BMPR2, unphosphorylated SMAD1/5/8, phosphorylated SMAD1/5/8 a and ID1, and increased activation of the BMP response element (Figures 4F and S5A). These findings demonstrate that SMURF1 inhibition augments BMP signaling in pulmonary vascular cells.

### SMURF1 inhibition re-establishes pulmonary vascular homeostasis

Modulation of BMP signaling, either through genetic or pharmacological manipulation, is known to alter key cellular phenotypes implicated in the pathology of PAH. BMP9 is the predominant BMP ligand that regulates pulmonary vascular endothelial homeostasis and acts protectively against vascular remodeling.^29^ Consistent with the identified increase in BMP signaling in human primary pulmonary artery endothelial cells (PAECs), we found that SMURF1 inhibition effectively reduced apoptosis and proliferation of these cells in the presence of a low BMP9 concentration (Figures 4G–4I). Similarly, in the presence of BMP4, the predominant ligand that regulates pulmonary vascular smooth muscle homeostasis, SMURF1 inhibition reduced proliferation and migration of primary PASMCs from patients with PAH (Figures 4J–4M). This effect was observed in the presence and absence of BMPR2 mutation.

To determine the on-target effects of allosteric SMURF1 inhibitors on the global proteome in a relevant disease-specific cellular context, PASMCs from patients with PAH were pre-treated with BMP4 under hypoxic conditions and exposed to the SMURF1 inhibitor or a vehicle (Figures S5B and S5C). Effective inhibition was demonstrated by increased protein levels of the established SMURF1 targets RhoA and TGFBR1 and key components of the BMP signaling pathway SMURF1, BMPR2, SMAD1, pSMAD1, and ID1 (Figure S5D).^60^^,^^61^ Gene set enrichment analysis showed that SMURF1 inhibition modulated multiple processes implicated in disease, including TGF-β superfamily (including BMP signaling) and interleukin-1β (IL-1β) signaling,^62^ extracellular matrix (ECM) remodeling, and fibrosis (Figure S5E).^63^

### SMURF1 inhibition reverses established experimental PAH pathology

**Figure 5:**
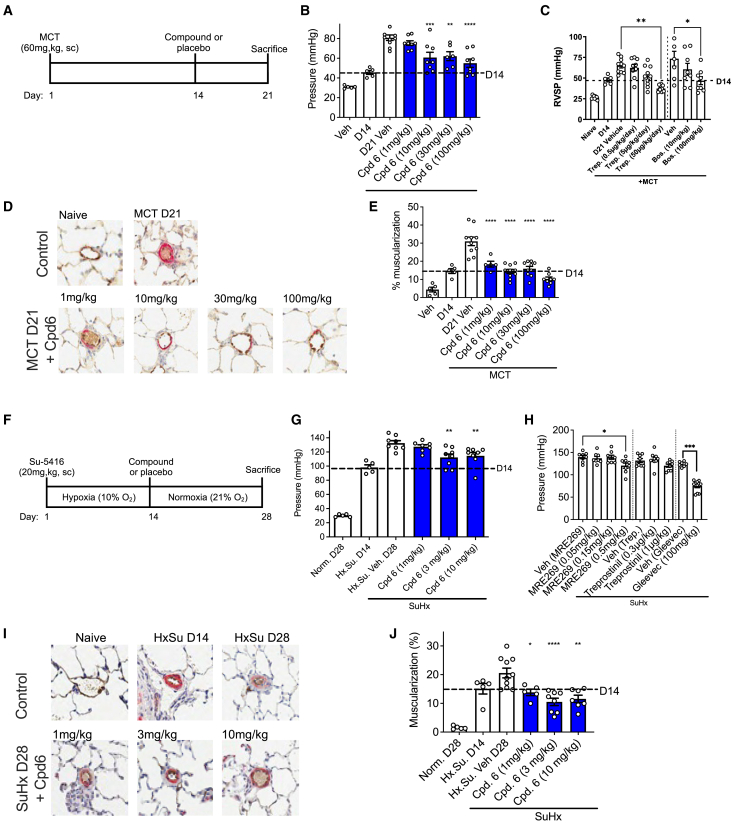
Inhibition of SMURF1 treats established experimental PAH (A) Experimental timeline for the monocrotaline study. (B–E) Right ventricular systolic pressure (RVSP) (B, SMURF1 inhibition; C, standard of care and experimental PAH therapies) and pulmonary vascular muscularization (D and E) are increased with disease and reduced by small-molecule inhibition of SMURF1 (*n* = 5–11). Histology (D): original magnification, x200. (F) Experimental timeline for the Su-5416 hypoxia study. (G–J) RVSP (G, SMURF1 inhibition; H, standard of care and experimental PAH therapies) and pulmonary vascular muscularization (I and J) are increased with disease and reduced by small-molecule inhibition of SMURF1 (*n* = 5–10). Histology (I): original magnification, x200. All bar graphs presented as mean ± SEM, ^∗^*p* < 0.05, ^∗∗^*p* < 0.01, ^∗∗∗^*p* < 0.001, ^∗∗∗∗^*p* < 0.0001, one-way ANOVA with Dunnett's correction.

Based on molecular and cellular data, we hypothesized that administering an allosteric SMURF1 inhibitor would restore BMP signaling in animal PAH models with reduced BMPR2 expression.^64^^,^^65^ Pulmonary hypertension was induced in Wistar Kyoto rats through treatment with monocrotaline (MCT) or SU5416 under hypoxic conditions (Figures 5A and 5F). 21 days after MCT administration and 28 days after the administration of SU5416 under hypoxic conditions (10% O_2_), right ventricular systolic pressure and pulmonary artery remodeling were increased (Figures 5B, 5C, 5G, and 5H). Consistent with the demonstrated augmentation of BMP signaling, allosteric inhibition of SMURF1 improved right ventricular systolic pressure (Figures 5B and 5G) and pulmonary artery remodeling (Figures 5D, 5E, 5I, and 5J) in a dose-dependent manner.

To assess the potential toxicity of allosteric SMURF1 inhibitors, a detailed biochemical and histopathology assessment was performed on blood and organs from the MCT study (Figures 5A–5E). No toxicologically relevant findings were observed (Table S2). Taken together, these data demonstrate that allosteric SMURF1 inhibitors effectively treated experimental PAH and exhibit minimal toxicity.

### Leveraging SMURF1’s allosteric mechanism to inhibit E6AP

**Figure 6:**
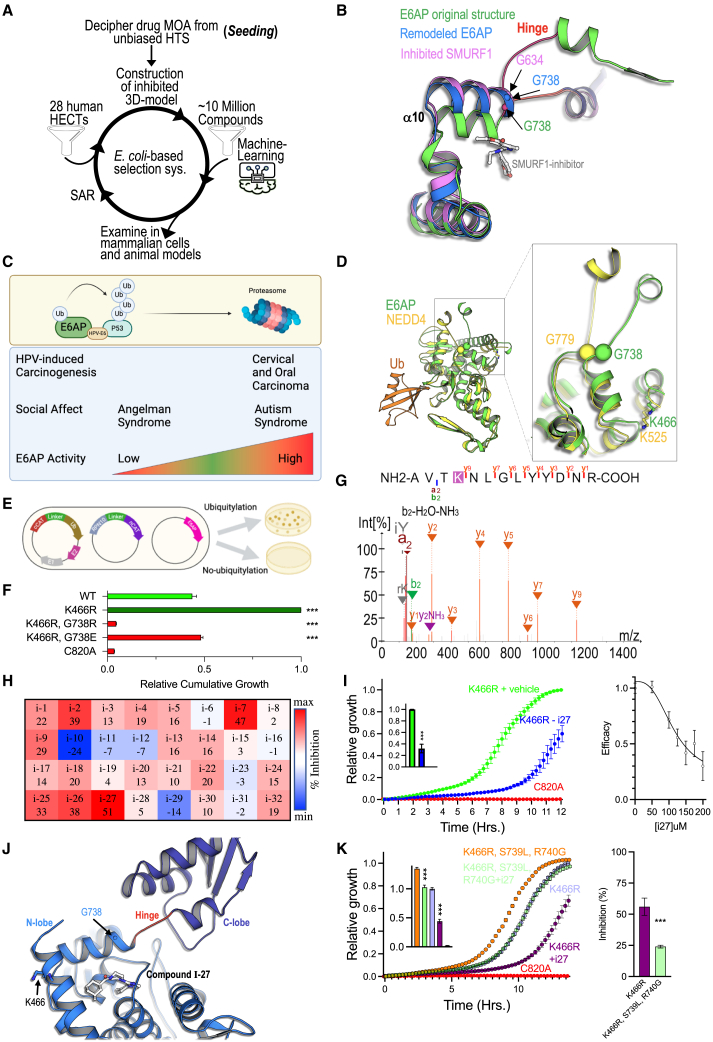
Structure-based identification of E6AP inhibitors (A) Schematic representation of the approach to hit identification. Remodeled HECT domain is formed by tethering on the inhibited SMURF1 structure and allosteric cryptic cavity used for an ML screen, followed by split-CAT validation. (B) Structure of the HECT glycine hinge and location of self-ubiquitylated lysine comparing the inhibited models of SMURF1 and E6AP and the active structure of E6AP. (C) Altered E6AP activity is associated with human disease. (D) Glycine 738 stems the hinge between the N- and C-lobes in E6AP with the lysine self-ubiquitylation target located at 466 (which corresponds to NEDD4 G779 and K525). (E) E6AP-dependent Rpn10 ubiquitylation split-CAT reporter system. (F) Activity of the constitutively active K466R E6AP is reduced by G738E mutation, demonstrating the importance of the glycine hinge and these residues. (G) Mass-spectrometry analysis showing self-ubiquitination of E6AP at K466. (H) Hit prioritization heatmap of 32 compounds derived from the ML-based screen and examined in the *E. coli* split-CAT system. Percentage inhibition is represented by a color scale from red (maximum inhibition) to blue (minimum inhibition). (I) Inhibition of E6AP with compound i-27 in *E. coli* split-CAT system and the dose-response curve for E6AP:i-27. (J) Predicted structural rearrangements of the E6AP glycine hinge in the presence of compound i-27. (K) Activity of the E6AP S739L, R740G escape (SMURF2lation) mutant in the presence of compound i-27. All studies, *n* = 3, mean ± SEM, ^∗∗^*p* < 0.01, ^∗∗∗^*p* < 0.001, one-way ANOVA with Dunnett’s correction or Student's t test as appropriate. See also Figures S2, S3, and S6.

As the SMURF1 inhibitor specifically restrains a conserved, essential motion between the N- and C-lobes of the HECT domain, we hypothesized that similar allosteric inhibition could be used as the basis of an *in silico* method for identifying potential inhibitors of other HECT E3 ligases (Figure 6A). To model a potential binding cavity, we threaded the amino acid sequence of HECT family members onto the structure of SMURF1 in its inhibited state (Figure 6B). The model was characterized by an elongated αH10 that traverses the conserved glycine residue, a shortened hinge between the N- and the C-lobes, and an open cavity in the N-lobe. We then employed ML-based screen to filter ∼8 million molecules, selecting candidates with the highest scores for downstream assessment.

To assess this approach and demonstrate its broad applicability, we investigated E6AP, the prototypic HECT E3 ligase. E6AP regulates levels of key cellular mediators, and dysregulation, induced by the human papillomavirus E6 protein, causes cervical and oropharyngeal cancers.^45^^,^^66^^,^^67^^,^^68^ E6AP is also critical to neurodevelopment, with increased or decreased activity resulting in human disease. Mutation of the conserved glycine in the αH10 of E6AP (G738E) reduces activity and causes Angelman syndrome.^45^ E6AP is expressed from the maternally imprinted UBE3A gene. Aberrant paternal expression, chromosomal duplication or triplication, or decreased protein degradation all increase E6AP levels and/or activity, leading to autism (Figure 6C).^69^^,^^70^

**Figure S6:**
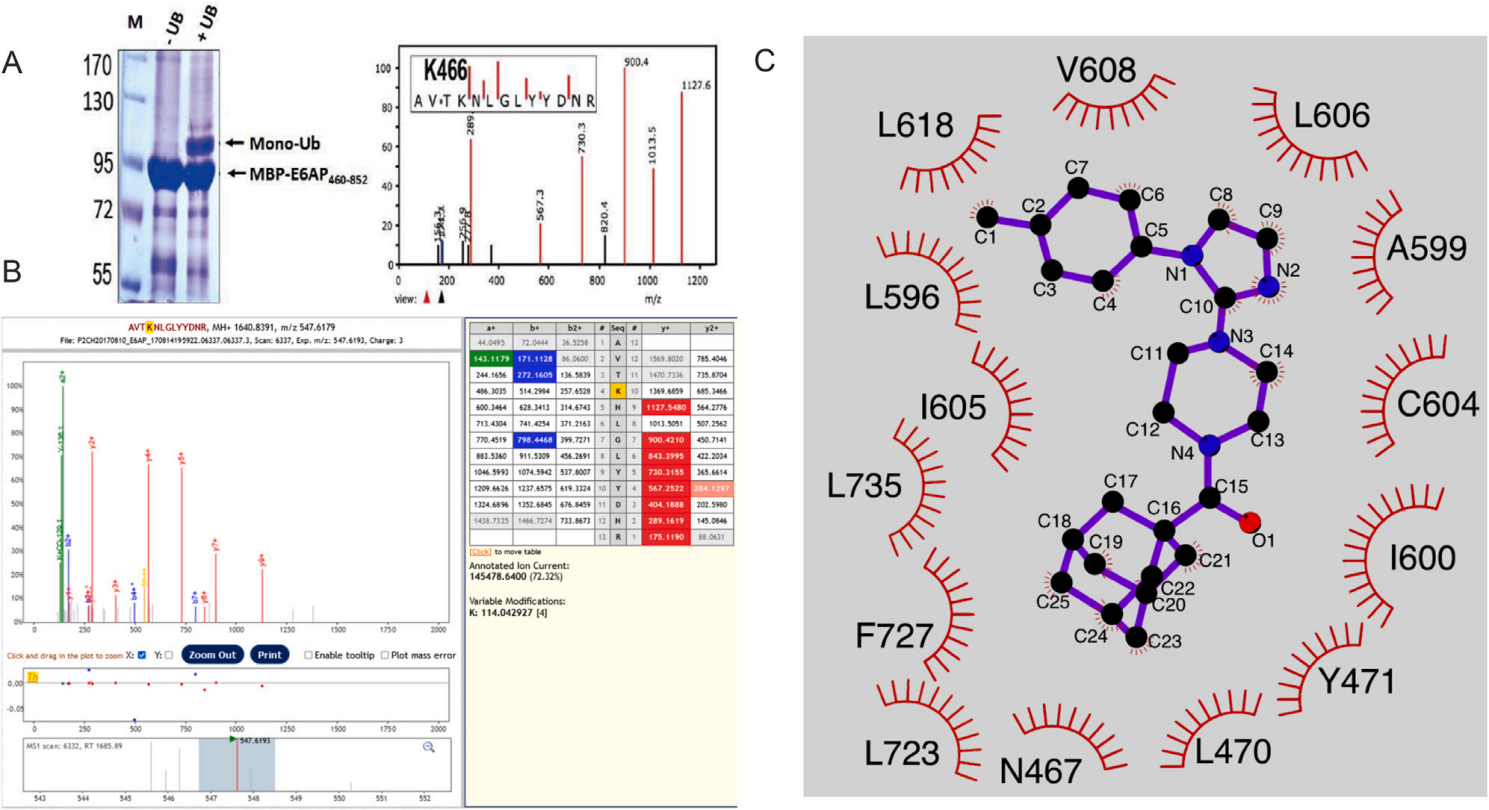
Self-ubiquitylation and inhibition of E6AP, related to Figure 6 (A) An E6AP self-ubiquitylation cascade was expressed in *E. coli* with or without ubiquitin. Protein was purified using nitrilotriacetic acid (NTA) affinity purification and run on SDS-PAGE, demonstrating self-ubiquitylation at K466. (B) In-gel analysis of the mass-spectrometry data. (C) LigPlot scheme of the E6AP:i27 interaction. Residues that form the cavity in E6AP and interact with the inhibitor i27 are presented. The chemical structure of the inhibitor (i-27): 1-[1-(4-methylphenyl)-imidazol-2-yl]-4-[adamantane-1-carbonyl]piperazine is shown at the center.

The AlphaFold model of full-length E6AP indicated that K466 spatially corresponds to K525 in human NEDD4, K438 in yeast Rsp5, and K381 in SMURF1. This lysine residue is critical to self-ubiquitylation-dependent oligomerization and subsequent inactivation (Figures 6D and S2F).^53^ Mutation of K466 is linked to autism and oropharyngeal cancer (E6AP-K466E: ClinVar: RCV002316869, and COSMIC: COSS1560555) and is predicted to constitutively hyperactivate the enzyme. To confirm the structural importance and demonstrate the capacity of the split-CAT reporter assay to detect alteration in E6AP-mediated ubiquitylation, we introduced a K466R mutation, which significantly increased ligase activity (Figures 6E and 6F). Further substantiating this finding, mass-spectrometry analysis, undertaken using a semi-tryptic peptide search approach to identify anticipated K-GG-modified peptides,^71^ revealed self-ubiquitylation at K466 of protein purified from *E. coli* expressing the full ubiquitylation apparatus of E6AP. This finding is consistent with re-analysis of datasets containing diGly peptides from human cell lines (HEK293 and U2OS), which demonstrated K466 ubiquitylation E6AP.^72^^,^^73^ Together these data support the concept of oligomerization-dependent inactivation of E6AP and provide a critical link to human disease (Figures 6G, S6A, and S6B).

The mutations G738E/R associated with Angelman syndrome^74^ (corresponding to SMURF1 G634) significantly decreased E6AP activity in the split-CAT system (Figure 6F). These two mutants provide, for the first time, structural mechanistic explanations for the E6AP mutations associated with cancer, autism, and Angelman syndrome.

We employed the split-CAT E6AP reporter system to select potential inhibitors from the *in silico* screening hits. Of 32 examined compounds, three demonstrated reduced E6AP activity by ≥39% (Figure 6H) without alteration of CAT activity or *E. coli* toxicity. Compound i-27 reduced E6AP activity in a dose-dependent manner (Figures 6I, 6J, and S6C), without affecting E1, E2, or CAT activities, suggesting selective inhibition. To determine if compound i-27 functions through the proposed allosteric mechanism, the Ser-Arg residues located immediately downstream of E6AP-G738 in the glycine hinge were substituted with the corresponding residues of SMURF2 (S739L, R740G). We found that the S739L, R740G double mutant did not reduce E6AP activity. In the presence of compound i-27, inhibition was reduced 2-fold, demonstrating escape from inhibition (Figure 6K). Together, these results confirm our hypothesis that computational screening for inhibitors that stabilize a remodeled, inhibited structure can identify compounds functioning through the same allosteric mechanism. Moreover, the described process of remodeling an inhibited cryptic cavity may provide a valuable strategy for discovering other allosteric inhibitors, including HECT ligases (Figure 6A).

## Discussion

To date, ∼3,000 distinct drugs have been approved for clinical use,^75^ yet they collectively target only 3% (∼650 proteins) of the ∼20,000 human gene products.^76^ Protein classes such as kinases, phosphodiesterases, ion channels, G-protein-coupled receptors, and nuclear hormone receptors account for the majority of targets due to the availability of druggable pockets. With an estimated 22% of the proteome being disease-modifying, there is a significant opportunity to expand the druggable space and identify new treatments for human diseases.^9^^,^^77^ Importantly, the 28 human HECT E3 ligases are attractive therapeutic targets without a confined druggable active-site pocket.^78^^,^^79^ This study provides an innovative approach that addresses the critical challenge of developing inhibitors for protein targets without an apparent active-site pocket. By applying a traditional, unbiased screening flowchart, we enabled the discovery of selective SMURF1 inhibitors that function via a novel allosteric mechanism. While computational methods based on existing structures have significantly advanced drug discovery, this study highlights the importance of traditional, unbiased approaches for identifying drugs for enzymes without described or obvious binding pockets, demonstrating the synergistic nature of these two strategies.

### The flexible (glycine) hinge: A critical, druggable protein structure?

Approximately 30% of human proteins contain a hinge region that enables critical motion between domains.^80^ Hinge flexibility requires amino acid residues with a high tolerance for Φ/Ψ dihedral angles, typically glycine and serine.^80^ In HECT domain proteins, limiting the essential motion between the N- and C-lobes is a natural regulatory mechanism of enzymatic activity. Different mechanisms can mediate this restriction, including action by the C2 domain of Nedd4 members,^9^^,^^51^^,^^52^ the WW domains,^81^ an α helix inserted between the lobes of the HECT domain,^82^ or oligomerization-dependent inactivation.^83^ The latter is achieved by various mechanisms, including ubiquitylation of a conserved lysine within the HECT αH1,^51^^,^^52^ which is observed across kingdoms.^49^^,^^50^ Interestingly, the E3-ligase SCF^FBXL15^ modulates BMP signaling through ubiquitylation of the conserved K381 on the αH1 of SMURF1,^84^ providing a naturally occurring mechanism for reversible regulation of a pathway linked to a number of human diseases, including PAH. Identifying HECT inhibitors that leverage a natural allosteric mechanism to limit hinge flexibility provides a framework for designing small molecules that modulate the function of other proteins dependent on hinge-mediated flexibility, including ion channels,^85^^,^^86^ immunoglobulins,^87^ and connexins.^88^^,^^89^

### Comparison to prior studies

Previous HTS have yielded allosteric inhibitors for E2 and E3 enzymes. The allosteric inhibitor CC0651 targets the E2 enzyme CDC34 and disrupts ubiquitin discharge and transfer to substrate proteins.^8^ SCF-I2, an allosteric inhibitor of the yeast E3 ligase CDC4, binds a cryptic pocket within the ligase's substrate receptor.^7^ However, residues forming the pocket are not conserved in the human ortholog FBXW7.^8^ These findings suggest that the dynamic nature of ubiquitylation enzymes holds potential for allosteric targeting that may be relevant to human disease.^6^

Several non-allosteric inhibitors of HECT E3s have been reported.^90^^,^^91^ HECLIN, originally identified in a screen of bicyclic peptides that compete with E2 binding to multiple nedd4-family members, was later found to induce oxidation of the catalytic cysteine via an unknown mechanism.^90^ A covalent modulator targeting a cysteine residue at the exosite ubiquitin-binding patch of NEDD4-1/2 prevents polyubiquitin chain formation. However, competing on ubiquitin binding to the exosite enhances the HECT ligases' activity and promotes monoubiquitylation.^51^^,^^52^^,^^53^

A key enabling feature of this study was HTS design and target validation. Previous efforts to identify E3-ligase inhibitors have used HTS assays that quantify ubiquitination of specific substrates^40^ or computational methods to identify small molecules that block substrate binding to the WW1 domain^39^ or to impede ubiquitin binding to the HECT domain^38^ and validated E3-target interactions in whole-cell systems. These approaches lack specificity, as the accessible motifs are present on multiple proteins, and ubiquitylation of a single target may be modulated by multiple E3s and DUBs. To simplify the HTS assay and downstream evaluation, we leveraged SMURF1 auto-ubiquitylation to develop a method that quantifies activity based on the accumulation of ubiquitylated SMURF1 and used the split-CAT system, with crystal structure-informed escape and susceptibility mutants, to provide a simple, dynamic readout of enzymatic activity without the need for purification or gel separation of components. Together, the detailed understanding of the mechanism of action permitted application to another HECT E3 ligase.

### Therapeutic HECT E3 inhibition for the treatment of human disease

The Mendelian association between mutations in the BMP/TGF-β superfamily and PAH provides a strong rationale for targeting related signaling pathways as a therapeutic strategy. Augmenting and/or modulating the BMP pathway has been shown to provide benefits in experimental models of PAH.^29^^,^^30^^,^^92^ Our data further implicate SMURF1 in disease pathology and demonstrate the therapeutic potential of SMURF1 inhibition. This finding is consistent with the current understanding of disease pathology and recent therapeutic advances in the field, where treatments that rebalance BMP/TGF-β pathways are under clinical development. Of these, an activin receptor IIa ligand trap has shown benefit in a late-phase clinical trial.^31^^,^^32^ Although SMURF1 mRNA is detectable across multiple organs, SMURF1 protein was only detectable in the lungs of patients with PAH. The tissue- and disease-restricted expression profile may explain the lack of toxicity observed in our studies of SMURF1 inhibition. Together, our data suggest that SMURF1 inhibition is an effective and potentially safe approach to restoring BMP signaling and improving vascular function in PAH.

A key differentiating feature of SMURF1 inhibition as a therapeutic approach is its ability to directly enhance the activity of the pathway receptor and downstream signaling mediators, which effectively addresses the receptor deficiency observed in humans. The development and availability of multiple therapeutic tools that modulate these pathways offer the opportunity to dissect disease pathology and mechanisms of therapeutic response through experimental medicine approaches, which may facilitate further therapeutic development and personalized therapy. Collectively, these observations and developments suggest that augmenting BMP signaling may provide a transformative, novel approach to the treatment of PAH.

A single ubiquitin ligase typically modulates multiple related protein targets through proteolytic and non-proteolytic mechanisms. These post-transcriptional modifications exert coordinated effects on cellular signaling and function. E6AP, the prototypic HECT E3 ligase, is central to the pathology of human papillomavirus (HPV)-related cancer, autism, Angelman syndrome, and other neurodevelopmental disorders.^93^^,^^94^ As we demonstrate in this study, the G738 mutation, which causes Angelman syndrome,^94^ leads to reduced protein activity through restriction of the glycine hinge. We also demonstrate modulation of E6AP activity through mutation of K466, an important site for self-ubiquitylation, mutation of which is associated with neurodevelopmental syndromes, providing, for the first time, a mechanistic explanation for the decrease or increase in ligase activity observed in human disease.

In summary, important implications of the reported findings include (1) the identification of a novel mechanism for the specific inhibition of HECT E3 ligase and potentially other glycine-hinge-containing proteins; (2) the identification of a novel inhibitor binding site through an unbiased biochemical screen, which would unlikely be achieved with *in silico* screens targeting putative candidate binding pockets; (3) the facilitated investigation of BMP pathway augmentation as a novel strategy for treating PAH through the specific inhibition of SMURF1; (4) a strategy for identifying and optimizing selective E3 ligase inhibitors with suitable drug-like properties offering clinical potential; (5) a novel insight into the importance of the E6AP glycine-hinge domain in HPV-related cancer, autism, and Angelman syndrome; and (6) a targeted approach to inhibit HECT E3 ligases, enabling faster identification of hits for lead optimization. To the best of our knowledge, this work introduces the first selective small molecular weight inhibitor of the SMURF1 HECT domain with demonstrated efficacy in animal models and suitable drug-like properties, offering an urgently needed therapeutic option for PAH and potentially other diseases involving SMURF1. The deciphered mechanism of action provides a new class of drugs for HECT E3 ligases and other glycine-hinge-containing proteins, opening a new druggable space with the promise of transformative therapies for diseases requiring innovative treatments.

### Limitations of the study

The *in vitro* and *in vivo* efficacy of inhibitors that do not act through this allosteric mechanism was not explored and could yield further insights into the regulation of HECT activity. Due to model efficacy, animal studies were undertaken on male rats, limiting the generalizability of findings; however, SMURF1 knockout improves right ventricular systolic pressure and pulmonary vascular remodeling in both male and female mice.^16^ Lung tissue was obtained from patients at the time of transplant, reflecting end-stage disease, which means that the expression profile of SMURF1 in an earlier stage of the disease is unknown. The low permeability, high efflux, and high expression of ligases in the *E. coli* system limit small-molecule inhibitor function; therefore, high concentrations of these molecules were required, possibly meaning some compounds were eliminated during the confirmation screen of E6AP inhibitors. E6AP has multiple targets and is associated with multiple diseases. It is therefore likely that a narrow inhibitor concentration range may be required.

## Resource availability

### Lead contact

Further information and requests for resources and reagents should be directed to and will be fulfilled by the lead contact David Rowlands (davidjrowlands8@gmail.com).

### Materials availability

The identified SMURF1 inhibitors described in this manuscript and non-standard assay reagents may be provided upon reasonable request to Novartis via material transfer agreement.

Split-CAT assay reagents may be provided upon reasonable request to Gali Prag via material transfer agreement.

Primary pulmonary artery endothelial and smooth muscle cells were provided by academic partners, and re-distribution is limited by collaboration agreements.

### Data and code availability

• Original data will be provided upon reasonable request. Study-specific crystal structures, code, and data have been deposited in an open repository.
• Full compound structures are described in our prior publication.^37^
• Standardized datasets. Accession numbers for crystal and proteomic datasets. Protein structures are available through the Protein Data Bank (https://www.rcsb.org/) under PDB codes: SMURF1—apo structure: 9FSK, SMURF1—compound 8 complex: 9FSJ, SMURF2—compound 8 complex: 9FSH.

## Acknowledgments

We thank Mark Southwood (Papworth Hospital Research Tissue Bank), the Novartis Laboratory Animal Services teams, Alessandro Piaia (Preclinical Safety), and René Lattmann, Robin Nathans, and Ritesh Tichkule (chemistry) at NIBR for the support that has facilitated the progression of the studies (Novartis, USA). A.M.K.R. was funded by the Wellcome Trust (CRCDF-206632/Z/17/Z), NIHR Research Clinical Lectureship, MRC (CRTF-MR/K002406/1), an Academy of Medical Sciences Starter Grant, and an Association of Physicians of Great Britain and Ireland Young Investigators Award. Research was carried out at the NIHR, Sheffield Biomedical Research Centre. A.M.K.R. is grateful to Richard Hughes, whose generous philanthropic support has helped to make this work possible. G.P. was funded by the Israeli Science Foundation (1440/21), the Israel Cancer Research Fund (ICRF) grant number 940283, and the Ministry of Science and Technology and DFKZ (Ca196). A.F. and A.K. are grateful for the Milner Fellowship support.

## Author contributions

A.M.K.R., S.G.-G., G.P., and D.J.R. conceived studies, acquired data, analyzed data, and wrote the manuscript. A.F., F.Z., C.Q., O.B., A.H., T.R., S.-M.H., D. Sutton, J.R., J.-H.Z., S.W., S.C., J.O., S.S., A.K., R.A., R.R., O.K., E.Y., T.T., S.K.B., R.E.J.B., J.S.C., N.S., N.O., and D. Shaw conceived studies, acquired data, analyzed data, and reviewed the manuscript. R.H. acquired data, analyzed data, and reviewed the manuscript. M.T. and R.J. conceived studies, analyzed data, and reviewed the manuscript. N.W.M. and M.H. acquired data, interpreted data, and reviewed the manuscript. M.J.T., G.J., and J.T. conceived studies, interpreted data, and reviewed the manuscript.

## Declaration of interests

D.J.R., S.G.-G., C.Q., O.B., R.H., A.H., T.R., M.T., R.J., D. Sutton, J.R., J.-H.Z., S.W., S.C., J.O., S.S., T.T., M.H., M.J.T., G.J., R.E.J.B., J.S.C., R.L., N.S., N.O., J.T., and D. Shaw are current or former Novartis employees. Two patents have been submitted related to this work: WO2020079687A1 (G.P.) and US11672782B2 (D. Shaw and N.S.).

## STAR★Methods

### Key resources table

**Table:** 

REAGENT or RESOURCE	SOURCE	IDENTIFIER
**Antibodies**		

SMURF1	Abnova	Cat#H00057154-M01; RRID:AB_566195
BMPR2	Biorbyt	Cat#orb69398
anti-myc (sc-40)	Sant Cruz Biotechnology	Cat#9E10; RRID:AB_2266850
SMAD1/5/9	Abcam	Cat#ab66737; RRID:AB_2192755
phospho-SMAD1/5	Cell Signaling Technology	Cat#9516S
ID1	CalBioreagents	Cat#M085; RRID:AB_1151763
GAPDH	Cell Signaling Technology	Cat#2118L
Fla-Tag (FG4R)	Thermo Fisher Scientific	Cat#MA1-91878; RRID:AB_1957945
Pan-Actin	Cell Signaling Technology	Cat#4968S
beta-actin	Cell Signaling Technology	Cat#4967
anti-α-SMA antibody	Sigma-Aldrich	Cat#A2547
anti-mouse NP antibody	Roche Diagnostics	N/A
anti-SMURF1 antibody	Abcam	Cat#AB57573; RRID:AB_945548
anti-mouse HRP-conjugated secondary antibody	Roche Diagnostics	Cat#760-150
anti-rabbit NP-conjugated antibody	Roche Diagnostics	Cat#760-4817
anti-vWF antibody	Agilent-Dako	Cat#A0082; RRID:AB_2315602
anti-α-SMA antibody	Sigma-Aldrich	Cat#A5228
anti-rabbit-HRP conjugated secondary antibody	Roche Diagnostics	Cat#460-4311
anti-mouse NP-conjugated secondary	Roche Diagnostics	Cat# 760-4816
anti-NP antibody	Roche Diagnostics	Cat#760-4827
anti-pSMAD1/5/8 antibody	Cell Signaling Technology	Cat#9511
HRP-conjugated polyclonal swine anti-rabbit secondary antibody	Dako-Agilent	Cat#E03553
isotype mouse IgG2a	Tonbo Biosciences	Cat#70-4724; RRID:AB_2621517
isotype rabbit IgG	Tonbo Biosciences	Cat#I-1000

**Bacterial and virus strains**		

T7 Express Competent E. coli	New England Biolabs	Cat#C2566H
Mach1T1R E. coli	Thermo Fisher Scientific	Cat#C862003

**Biological samples**		

Formalin-fixed, paraffin-embedded lung sections	Royal Papworth Hospital Research Tissue Bank	Research ethics: 08/H0304/56+5
Formalin-fixed, paraffin-embedded lung sections	Le Kremlin-Bicêtre, Paris, France	(CPP EST-III n°18.06.06)

**Chemicals, peptides, and recombinant proteins**		

GFP-tagged SMURF1	Origene	Cat#RG222902
GFP-SMRUF1 promoter	Genecopia	Cat#HPRM25572-PF02
BMP Response Element	Promega	Cat#pGL4[luc2P/hlD1/Hygro]
BMP4	R&D	Cat#314-BP-010
BMP9	R&D	Cat#3209-BP-010
annexin V	Promega	Cat#JA1000
Trypsin/Lys-C mix	Thermo Fisher Scientific	Cat#: A40009

**Stim Screen**		

human recombinant BMP2	R&D	Cat#355-BM-010
human recombinant BMP4	R&D	Cat#314-BP-010
human recombinant BMP6	R&D	Cat#507-BP-020
human recombinant BMP7	R&D	Cat#354-BP-010
human recombinant BMP9	R&D	Cat#3209-BP-010
Recombinant Human TGF-beta 1 Protein	R&D	Cat#7754-BH-005
Recombinant Human TRAIL/TNFSF10 Protein	R&D	Cat#375-TL-010
Recombinant Human Osteoprotegerin/TNFRSF11B Protein	R&D	Cat#6945-OS-025
Recombinant human FGF	R&D	Cat#233-FB-025
Recombinant Human IL-1 beta/IL-1F2 Protein	R&D	Cat#201-LB-005
Recombinant Human IL-6 (HEK293-expressed) Protein	R&D	Cat#7270-IL-025
Recombinant Human IL-13 Protein	R&D	Cat#213-ILB-005
Recombinant Human IL-18/IL-1F4 Protein	R&D	Cat#9124-IL-010
Recombinant Human CX3CL1/Fractalkine Protein	R&D	Cat#365-FR-025
Recombinant Human TNF-alpha Protein	R&D	Cat#210-TA-020
Recombinant Human IFN-alpha A (alpha 2a) Protein	R&D	Cat#11100-1
Recombinant Human IFN-gamma Protein	R&D	Cat#285-IF-100
Recombinant Human PDGF-BB Protein	R&D	Cat#220-BB-050
Recombinant Human EGF Protein	R&D	Cat#236-EG-200
H2L 5765834	R&D	Cat#4870/10
Recombinant Human CTGF/CCN2 Protein, CF	R&D	Cat#9190-CC-050
Recombinant Human HGF Protein	R&D	Cat#294-HG-005
Recombinant Human VEGF	R&D	Cat#293-VE-010
Serotonin hydrochloride	Tocris	Cat#3547
Endothelin-1	Tocris	Cat#1160
DETA NONOate	Tocris	Cat#6077
VIP	Tocris	Cat#1911/1
Bosentan	Sigma	Cat#SML1265-10MG
Riociguat	Sigma	Cat#B8810-5MG
Sildenafil	Simga	Cat#PZ0003-5MG
U46619	Simga	Cat#D8174-1MG
FK506	Sigma	Cat#F4679-5MG
Rosiglitazone	Sigma	Cat#R2408-10MG
MRE-269	Cayman Chem	Cat#10010412-5MG
Bafilomycin A1	Alfa Aesar	Cat#J67193

**Critical commercial assays**		

PathHunter Prolabel detection kit	DiscoverX	Cat#93-0180L
Bright-Glo Luciferase Assay System	Promega	Cat#E1500
Maxiprep kit	Qiagen	Cat#12162
Lipofectamine LTX	Thermo Fisher Scientific	Cat#A12621
Lipofectamine 3000	Thermo Fisher Scientific	Cat#L3000001
Protein Simple Western Blot System (Wes)	ProteinSimple	Cat#004-600
Micro BCA Protein Assay kit	Thermo Fisher Scientific	Cat#23235
TMT11plex Isobaric Label Reagent kit	Thermo Fisher Scientific	Cat#A34808
RIPA lysis and extraction buffer	Thermo Fisher Scientific	Cat#89900
Pierce 660 nm Protein Assay Reagent	Thermo Fisher Scientific	Cat#22660
BSA Protein Assay Standards set	Thermo Fisher Scientific	Cat#23208

**Deposited data**		

Code	This paper	https://github.com/Novartis/px_tmt_daa
ProteomeXchange Consortium via the PRIDE partner repository	Kathman et al.^91^	PRIDE: PXD051134
Protein structures deposited in the Protein Data Bank (https://www.rcsb.org/):	This paper	PDB: SMURF1 - apo structure: 9FSK; SMURF1. PDB: compound 8 complex: 9FSJ. PDB: SMURF2 - compound 8 complex: 9FSH

**Experimental models: Cell lines**		

Mammalian HEK293 cells	ATCC	Cat#CRL-1573
primary human pulmonary arterial smooth muscle cells	Royal Papworth Hospital Research Tissue Bank	research ethics: 08/H0304/56+5
primary human pulmonary arterial endothelial cells	Lonza	Cat#CC-2530

**Experimental models: Organisms/strains**		

Wistar Kyoto rats (WKY/NHsd)	Harlan (now Inotiv)	Cat#023
SU5416	Sigma	Cat#S8442
Crotaline	Sigma	Cat#C2401

**Oligonucleotides**		

ACVRL1 (siRNA)	Thermo Fisher Scientific	Assay ID: VHS41062, Cat#1299001
BMPR2 (siRNA)	Thermo Fisher Scientific	Assay ID: S2044, Cat#4390824
ENG (siRNA)	Thermo Fisher Scientific	Assay ID: S4677, Cat#4392420
SMAD9 (siRNA)	Thermo Fisher Scientific	Assay ID: S8415, Cat#4392420
SMURF1 (siRNA)	Thermo Fisher Scientific	Assay ID: S32798, Cat#4390824
SMURF1 qPCR probe	Thermo Fisher Scientific	Hs00410929_m1

**Software and algorithms**		

DiscoverX PathHunter™	Eurofins	N/A
COOT	Global Phasing Ltd.	N/A
BUSTER	Global Phasing Ltd.	N/A
PyMOL	DeLano Scientific LLC	N/A
Incucyte S4 live cell imager	Sartorius	N/A
ProteomeDiscoverer (PD) ver. 2.4	Thermo Fisher Scientific	N/A
clusterProfiler	Mund et al.^90^	N/A
MetabaseMap	GeneGO, Clarivate	N/A
MSigDB	Broad Institute, Cambridge	N/A
ImagePro	Media Cybernetics	N/A
HHblits	Max Planck Institute	N/A

### Experimental model and study participant details

#### Bacteria

*E. coli* and Mach1T1R *E. coli* cells were cultured in LB (agar or liquid) at 37^o^C.

#### Cell lines

Mammalian HEK293 cells were obtained from ATCC (CRL-1573).

Mammalian HEK293 T-REx cells were obtained from Invitrogen (R71001).

Primary human pulmonary arterial smooth muscle cells from patients with PAH undergoing transplant were obtained from the approved tissue bank at Royal Papworth Hospital Research Tissue Bank, UK (Research ethics: 08/H0304/56+5).

Primary human pulmonary arterial endothelial cells were obtained from Lonza (CC-2530).

#### Rats

All studies described in this report were performed according to the UK Animals (Scientific Procedures) Act 1986 under project license number: 70/7182.

##### Monocrotaline model

Animals (male Wistar Kyoto rats, Harlan, UK) were injected with subcutaneous monocrotaline (Crotaline, Sigma, Poole, UK) at a dose of 60 mg/kg (0.5 ml/kg).^16^ On day 14, animals were dosed with 0.5 ml test agent or vehicle orally (p.o.) for 7 days either once or twice per day as indicated.

##### Sugen/hypoxia model

Animals (male Wistar Kyoto rats, Harlan, UK) were injected with a subcutaneous Sugen (SU5416, Sigma, Poole, UK) at a dose of 20 mg/kg (1 ml/kg) before being caged in hypoxic conditions (10% O_2_, normobaric with constant humidity and CO_2_ levels, 24 hours/day) for 14 days.^16^ Animals were then returned to normoxia (21% O_2_) and test agents were administered via oral gavage (0.5 ml) once daily for a further 14 days. All SMURF1 inhibitors were prepared in 0.5% methyl cellulose, while 0.5% Tween 80 and imatinib were prepared in sterile water. At study end, animals were anesthetized using ketamine/medetomidine, the jugular vein was surgically exposed and blood flow was isolated with a distal ligature. A small hole was made in the vessel and a 2F Millar pressure/volume catheter was introduced and progressed into the right ventricle (RV), where an average RV pressure was measured during systole (RVSP). The lungs were excised from the rats and inflated with 10% neutral-buffered formalin and immersed in the same solution for 24–48 hours to complete fixation.

#### Human subjects

Histological lung samples from 33 patients with PAH (20 female and 13 male, 3 hereditary PAH (BMPR2 mutation), 20 idiopathic PAH, 7 congenital heart disease associated PAH, 3 connective tissue disease associated PAH) and 19 patients with an alternate diagnosis (10 male and 9 female) were obtained from approved tissue banks at Royal Papworth Hospital Research Tissue Bank, UK (Research ethics: 08/H0304/56+5) and Le Kremlin-Bicêtre, France (CPP EST-III n°18.06.06). All patients gave informed consent.

### Method details

#### Biochemical SMURF1 high-throughput biochemical assay and E3-ligase selectivity panel

The SMURF1 high-throughput screen (HTS) was conducted as a fluorescence resonance energy transfer (FRET) assay, where the donor lanthanide fluorophore is europium, encapsulated by cryptate for stability, and the acceptor fluorophore is a cross-linked allophycocyanin (XL665).^95^ When both fluorophores are in proximity through the ubiquitylation reaction, the excited europium cryptate emits light at 590 nm, and the energy is transferred to XL665, which releases a fluorescence signal at 665 nm. In the primary screen, the polyubiquitylation of SMURF1 was detected with a mixture of wild-type europium-labeled ubiquitin and biotinylated ubiquitin, which elicited a signal upon the addition of streptavidin labeled XL665 (Figure S2). For validation screening, the interaction between the glutathione S-transferase (GST)-tagged SMURF1 and the mixture of wild-type ubiquitin and europylated ubiquitin elicited a signal that was proportional to the level of polyubiquitylation upon the addition of anti-GST XL665 (Figure S2). A ratiometric readout of 665 nm/590 nm was taken to reduce some of the assay variation. The experimental conditions of the HTS correspond to the SMURF1 assay described in detail for the selectivity panel.

#### E3-ligase selectivity assay panel

E3-ligase specificity assay panel employed the same time-resolved (TR)-FRET principle. Compounds were tested for HECT E3-ligase selectivity in an assay panel consisting of the following ubiquitin E3-ligases: SMURF1 (aa 119-757), SMURF2 (aa 251-748), WWP1 (aa 546-922), WWP2 (aa 494-870), Nedd4 (aa 619-1000), Nedd4L (aa 693-1074), Itch (aa 433-903) and E6AP (aa 2-852). All E3-ligases were expressed as N-terminal hemagglutinin (HA)-tagged fusion proteins in *E. coli*, except for full-length E6AP which was expressed in baculovirus-infected insect cells as a N-terminal GST-fusion protein.

For compound testing, serial dilutions were prepared in DMSO and 50 nl was transferred to the assay plates (Greiner 384-Well Small Volume Plate, white, 30 μl wells, PS; Greiner Bio-One, #784075). To each well of these assay plates, 4.5 μl E3-ligase solution was added, followed by 4.5 μl of the pre-incubated mix containing E1a, E2 and biotinylated ubiquitin mix or the pre-diluted ubiquitin (control) in 50 mM HEPES, pH 7.5, 50 mM NaCl, 10 mM MgCl_2_, 20 μM ATP, 0.1 mM DTT, 0.002% Triton X-100. The final assay concentrations of the components were 3–30 nM E3-ligase, 20 nM E1a, 50 nM E2 (UbCH5b for SMURF1/2, UbCH7 for Itch and E6AP, UbCH5c for WWP1/2 and Nedd4/4L) and 20 nM biotinylated ubiquitin or the unmodified ubiquitin (control).

After 45 minutes of incubation at room temperature, the ubiquitylation reactions were stopped by adding 4.5 μl STOP solution (2 mM NEM, 50 mM HEPES, pH 7.5, 150 mM NaCl, 0.01% Triton X-100), immediately followed by adding 4.5 μl of detection solution, containing the XL665-labeled antibody and the streptavidin-coupled europium (20 nM streptavidin europium, 40 nM XL665-labeled anti-HA or anti-GST antibody, 50 mM HEPES, pH 7.5 150 mM NaCl, 0.01% Triton X-100), to a total volume of 18.0 μl. After a further incubation of 45 minutes at room temperature in the dark, the plates were transferred to a Pherastar fluorescence reader (BMG Labtech, Germany) to measure the TR-FRET signal. A ratiometric readout of 665 nm/590 nm was taken to reduce some of the assay variation.

#### Cellular compound screening assay to assess SMURF1 inhibition

The DiscoverX PathHunter™ (Eurofins, USA) technology was used to determine Prolabel-tagged SMURF1 levels in an enzyme complementation assay of the cell lysate. HEK293 T-Rex cells (Invitrogen) were stably transfected with SMURF1, fused at the N-terminus with the Prolabel tag (a fragment of beta-galactosidase), which complements the exogenously added enzyme fragment to form functional beta-galactosidase. Upon incubation with small molecules, a chemiluminescent signal is generated by the addition of a substrate to the cell lysate. The signal is proportional to SMURF1 levels (Figure S2).

To assess SMURF1 inhibition and subsequent stabilization, a stable HEK293 cell pool expressing a Prolabel-SMURF1 fusion protein was generated via lentiviral transduction. To prepare the virus, the Prolabel-tagged SMURF1 construct was inserted into the p40 lentiviral vector conferring puromycin resistance. HEK293 cells were seeded into a 6-well plate at 2x10^5^ cells per well the day before infection. On the next day, the medium was exchanged with fresh high-glucose Dulbecco's Modified Eagle's Medium (DMEM), 10% fetal bovine serum (FBS; (Cat. # 10500064, Thermo Fisher Scientific, Gibco, UK) and penicillin-streptomycin (pen/strep) with 10 μg/ml polybrene. Then, 100 μl of the ProLabel-SMURF1 lentivirus preparation was added to the medium. Cells were incubated overnight, after which the medium was replaced by fresh polybrene-free medium for 24 hours. Thereafter, the medium was replaced with fresh medium containing puromycin (2 μg/ml final concentration) to start the selection of clones with integrated SMURF1. The cells were incubated in a selection medium until all cells in the untransfected control plate had died. The puromycin-resistant HEK293_Prolabel hSMURF1 cells were expanded in a puromycin-containing selection medium and frozen in aliquots for further use.

For compound testing, serial dilutions were prepared in DMSO and 250 nl was transferred to the assay plates (Greiner 384-well plates, PS, white, CELLSTAR, uClear). Cells were detached from the flask after a short incubation with trypsin-EDTA, counted and diluted to a concentration of 0.75x10^6^ cells/ml in a culture medium without puromycin. The expression of SMURF1 was induced by adding doxycycline to a final concentration of 0.2 μg/ml. A volume of 20 μl of the cell suspension were seeded into the compound plates by using the MULTIDROP 384. The plates were incubated overnight at 37°C with 5% CO_2_. Thereafter, the levels of SMURF1 were determined using the PathHunter™ Prolabel detection kit (Cat#: 93-0180L, DiscoverX). First, 10 μl of a lysis/CL detection working solution was added, followed by the addition of 5 μl enzyme acceptor EA. The plates were mixed on a plate shaker and incubated for 2-3 hours at room temperature before measuring the chemiluminescent signal in the PheraStar plate reader for 1 second per well.

#### Crystallography

Purified SMURF1_377-751_ was crystallized using the hanging drop vapor diffusion method. A volume of 1 μL SMURF1 at a concentration of 15.1 mg/ml in 50 mM Tris pH 8.0, 200 mM NaCl, 2 mM TCEP and 10% glycerol were mixed with 1 μL reservoir solution (25 % PEG 3350, 0.1 M Bis Tris pH 5.9, 0.2 M MgCl_2_, 0.02 M NH_4_OAc) and equilibrated against 600 μL reservoir solution.

Purified SMURF2_251-748_ was crystallized by the hanging drop vapor diffusion method. A total of 2 μL SMURF2 at a concentration of 10.7 mg/ml in 50 mM Tris pH 8.0, 20 mM NaCl and 2 mM TCEP was mixed with 1 μL reservoir solution (1.5 M NaH_2_PO_4_/K_2_HPO_4_ pH 6.8, 100 mM NaOAc) and equilibrated against 1 mL of reservoir solution. Crystals were soaked by the addition of 0.5 μL inhibitor stock solution (100 mM inhibitor in 90% DMSO) to the crystal-containing drop for 30 minutes.

Co-crystals of SMURF1 in complex with compound 8 were grown using the sitting drop vapor diffusion method. A volume of 0.2 μL SMURF1 at a concentration of 15.1 mg/mL SMURF1 in 50 mM Tris pH 8.0, 20 mM NaCl, 2 mM TCEP, 2 mM compound 8 and 1.8% DMSO was mixed with 0.3 μL reservoir solution (25% PEG1500, 100 mM SPG pH 9) and equilibrated against 80 μL reservoir solution.

For data collection, crystals were flash-cooled in liquid nitrogen. X-ray diffraction data were collected from single crystals at the Swiss Light Source, beamline X06DA equipped with a MAR225CCD detector for SMURF1 and beamline X10SA equipped with a Pilatus Pixel detector for crystals of SMURF1 and SMURF2 in complex with compound 8. The diffraction data were processed and scaled with the autoPROC toolbox.^96^ The structures were solved by molecular replacement using the coordinates of PDB code 1zvd as the search model and the program MOLREP.^97^ For SMURF1 structure solution, the search model was split in the C- and N-lobe and the hinge residues and the helix N-terminal to the hinge were omitted from the search model. The software programs COOT^98^ and BUSTER (Global Phasing Ltd. Cambridge, UK) were used for iterative rounds of model building and structure refinement. Images were generated using the program PyMOL (DeLano Scientific LLC, San Carlos, CA, USA). PDB codes: SMURF1 - apo structure: 9FSK, SMURF1 - compound 8 complex: 9FSJ, SMURF2 -compound 8 complex: 9FSH.

#### Bacterial E3-ligase assay (split-CAT assay for ubiquitin ligase activity)

Assays were performed as previously described.^49^^,^^50^ The selection plasmids were co-transformed into Mach1 *E. coli* cells. Growth assays were performed in LB agar or liquid at 37^o^C. Chloramphenicol (CAM) concentrations were optimized according to the largest growth shift between the hyperactive mutant to the catalytic dead mutant in SMURF1 (ΔC2-K381R; ΔC2-C725A) or E6AP (HECT-K466R; HECT-C820A) for each assay as previously described.^48^^,^^50^ Following inoculation, bacteria were grown to OD_600_ 0.2-0.4 prior to CAM administration. After an alarm, upon returning from the safe zone, OD_600_ was measured. Fast growing strains were attenuated by refrigeration at 4 ^o^C and OD_60_ adjusted to account for potential overgrowth.

We constructed a split-CAT-based E3-dependent Rpn10 full-length ubiquitylation cascade to monitor the effect of inhibitor and point mutations in ubiquitin E3-ligases. Specifically, yeast Rpn10 was tethered to N-CAT as a general ubiquitylation target and co-expressed with C-CAT-Ub, E1, E2 and either human SMURF1_235-757_ (wild-type ΔC2, K381R or catalytic dead C725A) or E6AP_453-852_ (wild-type HECT, K466R or catalytic dead HECT-C820A).

To assess the ubiquitylation of BMPR2 and SMAD1, we substituted Rpn10 with BMPR2_174-558_ cytosolic portion or SMAD1 (full length) fused to the N-CAT. To further characterize the mechanism of action of SMURF1 inhibitors, we used the hyperactive ΔC2-K381R mutant of SMURF1 as a platform for other mutations in the ligase (G634E/P, G637ΔKID, 633GGLD>CGLG and insertion of GGLD downstream to D636) and tested their activity, with Rpn10 serving as a general ubiquitylation target. Accordingly, we used the hyperactive mutant E6AP HECT-K466R for other mutations in the ligase (G738E/R, 737CGSR>CGLG). We used the phospho-mimetic mutant (S210,214E) of SMAD1_206-236_, as a specific target for SMURF1 mutants (N507A, D636G and R686A) and the SMURF2 mutant (G630D). Inhibition assays were performed in liquid LB using a 96-well plate with Tecan Sunrise reader. The conditions included high-intensity shaking for 9.5 minutes and resting for 10 minutes, followed by a 5-minute shake and read at 595 nm. Growth was monitored between 12 and 36 hours, as indicated.

#### Cellular assay to determine SMURF1 stability and promoter expression using GFP-tag

To measure SMURF1 protein abundance and promoter activity, stable HEK293 cell lines were generated using a vector encoding: 1) SMURF1 protein with C-terminal green fluorescent protein (GFP) (RG222902, Origene); 2) the SMURF1 promoter and a GFP sequence (HPRM25572-PF02, Genecopia). HEK293 cells were transformed as per standard protocols. Briefly, after plasmid preparation with Maxiprep kit (Qiagen), cells were transfected with 2.5 μg of plasmid DNA using 1, 1.5 and 2 μL of Lipofectamine LTX (Thermo Fisher Scientific, USA) as per the manufacturer’s instructions and GFP fluorescence was measured with Incucyte S4 Live-Cell Imager (Sartorius, Germany). Optimal transfection conditions were determined by maximal GFP fluorescence. After sorting, cells were seeded and cultured for 4-6 weeks with appropriate antibiotic selection. GFP fluorescence was measured against time on the Incucyte S4 Live-Cell Imager and the signal after 12-hour compound incubation was quantified as an experimental endpoint.

##### siRNA knock-down

For RNA knock-down, siRNA was delivered using Lipofectamine 3000 (Thermo Fisher Scientific, USA) and the following probes as per the manufacturer’s instructions (Thermo Fisher Scientific, USA): *ACVRL1* (Assay ID: VHS41062, Cat#1299001), *BMPR2* (Assay ID: S2044, Cat#4390824), *ENG* (Assay ID: S4677, Cat#4392420), *SMAD9* (Assay ID: S8415, Cat#4392420), SMURF1 (Assay ID: S4677, Cat#4392420).

#### HEK over expression assays

HEK293 cells were seeded at 3x10^5^ cells per well in a 12-well plate and cultured in DMEM with GlutaMAX™ (Cat. # 10566-016, Thermo Fisher Scientific, Gibco, UK), supplemented with 10% FBS and 1% v/v of 100X antibiotic-antimycotic reagent (pen/strep; Cat. # 15240062, Thermo Fisher Scientific, Gibco, UK) overnight at 37°C in 5% CO_2_. The next day, the medium was replaced by a 1 mL growth medium without antibiotics containing SMURF1 inhibitor (compound 6, 5μM). Cells were then transfected with plasmids expressing SMURF1 (wild type or mutant), the target (SMAD1 or BMPR2) and ubiquitin using Lipofectamine™ 3000 Transfection Reagent (#L3000015, Invitrogen), according to the manufacturer’s instructions. Protein was extracted using RIPA lysis and extraction buffer (Cat. # 89900, Thermo Fisher Scientific, UK), along with protease and phosphate inhibitor cocktail (Cat. # 78444, Thermo Fisher Scientific, UK). Protein concentration in the samples was determined using the Pierce 660 nm Protein Assay Reagent (Cat. # 22660, Thermo Fisher Scientific, UK) and the Bovine Serum Albumin (BSA) Protein Assay Standards set (Cat. # 23208, Thermo Fisher Scientific, UK) prior to Western blot analysis.

#### PASMC assays

Primary human pulmonary arterial smooth muscle cells (PASMCs) from patients with PAH undergoing transplant were obtained from approved tissue banks in Royal Papworth Hospital Research Tissue Bank, UK (Research ethics: 08/H0304/56+5).

##### Proliferation

Cells were seeded at 5,000 per well in 96-well plates (3595, Corning, USA) in full growth media (DMEM with 10% low Ig FBS and 5 ml of pen/strep, Gibco, USA), allowed to adhere and then swapped into starvation media (DMEM with 0.2% low Ig FBS) for 24 hours to synchronize the cells in a non-proliferative state.^16^ Cells were cultured in the indicated concentration of growth media and stimulated with BMP4 (314-BP-010, R&D, USA) with increasing concentrations of SMURF1 inhibitor to determine the effect on cellular proliferation; phase microscopy was used to determine cell confluence on the Incucyte S4 Live-Cell Imager with packaged analysis software. Confluence was determined along the entire time course by expressing total cell numbers per well to the maximum cell number obtained by culturing cells in full-growth media.

##### Migration

Cells were seeded at 3,000 per well in 96-well plates in full growth media (DMEM with 10% low Ig FBS and 5ml of pen/strep, Gibco) and allowed to adhere. Migration, in the presence of indicated growth media, stimulant (BMP4, 10 ng/ml final, 314-BP-010, R&D) and the compound was assessed by disc closure as per the manufacturer’s instructions (CBA-126, Cell Biolabs) using phase microscopy to determine migration on the Incucyte S4 Live-Cell Imager with packaged analysis software. Disc size was determined 4, 8, and 12 hours after removal of the biocompatible hydrogel.

##### Cell signaling studies

Cells were seeded at equal densities, allowed to adhere and then incubated in starvation media for 24 hours to synchronize cells in a non-proliferative state in the presence of DMSO or 5 μM SMURF1 inhibitor. Cells were stimulated with a concentration of BMP4 (10 ng/ml) found to elicit a sub-maximal response in the absence of SMURF1 inhibitor to initiate signaling. Two hours after the addition of BMP4, cells were harvested and lysed.

#### PAEC assays

Primary human pulmonary arterial endothelial cells (PAECs) (CC-2530, Lonza, USA) were seeded in 96-well plates (3570, Corning, USA) at 5,000 per well in full-growth media (CC-3162, Lonza, USA) and allowed to adhere.

##### Apoptosis

16 hours before the addition of an apoptotic stimulus, PAECs were transferred into EBM-2 basal media (Lonza, USA) with 2% FBS, 100 U/ml penicillin, 100 mg/ml streptomycin and 0.25 mg/ml amphotericin B in the presence of indicated growth media, stimulant (BMP9, 10 ng/ml final, 3209-BP-010, R&D, USA) and compound/DMSO.^29^ Following incubation, cells were either left unstimulated or treated with 10 ng/ml TNFα and 20 μg/ml cycloheximide to induce apoptosis, which was assessed by the presence of annexin V as per the manufacturer’s instructions (JA1000, Promega, USA).

##### Proliferation

Cells were seeded at 5,000 per well in 96-well plates in full-growth media and allowed to adhere.^29^ Cells were cultured in the indicated concentration of growth media and stimulated with BMP9 (3209-BP-010, R&D, USA) with increasing concentrations of SMURF1 inhibitor to determine the effect on cellular proliferation using phase microscopy to determine cell confluence on the Incucyte S4 Live-Cell Imager with packaged analysis software. Confluence was determined along the entire time course by expressing total cell numbers per well to the maximum cell number obtained by culturing cells in full-growth media.

#### Western blot

Specific proteins were quantified using the Protein Simple Western Blot System (Wes) (ProteinSimple, CA, USA), gel electrophoresis imaged on the Odyssey SA imaging system (LI-COR Biosciences) or Fx7 imager, as previously described.^16^ The following primary antibodies were used and paired with appropriate secondary antibodies for detection: SMURF1 (H00057154-M01, Abnova, USA and H60, Sant Cruz Biotechnology, USA), BMPR2 (orb69398, Biorbyt, UK and BD, #612292), anti-myc (sc-40, Sant Cruz Biotechnology, USA), SMAD1/5/9 (ab66737, Abcam, USA), phospho-SMAD1/5 (9516S, Cell Signaling Technology, USA), ID1 (M085, CalBioreagents, USA), GAPDH (2118L, Cell signaling Technology, USA), Fla-Tag (FG4R, Thermo Fisher Scientific, MA1-91878), Pan Actin (4968S, Cell Signaling) and beta-actin (4967, Cell Signaling Technology, USA).

#### PCR expression studies

Human PASMCs from patients with PAH were treated with agents that are associated with PAH, including cytokines, chemokines, growth factors and small molecule pathway probes, and cultured to identify modulators of SMURF1 mRNA measured by PCR as per manufactuers instructions (Hs00410929_m1, ThermoFisher, *n*=2 donors, triplicate measures).

#### Luciferase BMP signalling assay (BMP response element)

PASMCs were transfected with 100 ng of plasmid vector (pGL4 luc2p/ID1/Hygro, Promega pRL-TK, Promega). Cells were stimulated with BMP4 (R&D Systems) at 50 ng/ml in DME medium containing PSA solution (Invitrogen) in 0.2% fetal bovine serum and treated as indicated. Twenty-four hours after transfection, cells were harvested and lysed and luciferase reporter activities measured using the Dual-Glo Luciferase Assay System (Promega). Firefly luciferase activity was normalized to Renilla luciferase activity.

#### Proteomic analysis

PASMCs from PAH patients carrying either mutant BMPR2 (C347Y) or wild-type allele were cultured to 80–90% confluence and then swapped into starvation media for 24 hours to synchronize cells in a non-proliferative state in the presence of DMSO or SMURF1 inhibitor compound 6 (5 μM) in normoxic or hypoxic conditions. Hypoxic cells were cultured in 1% O_2_ at 37°C for 5 days prior to cell harvest. All media changes were performed with media that had been incubated in hypoxic conditions for 24 hours to ensure no re-oxygenation. Cells were stimulated with BMP4 (10 ng/ml) to initiate signaling and prior to cell harvest for protein isolation and quality control by quantification via the Protein Simple Western Blot System.

Treated PASMCs were collected, washed 3 times with ice-cold PBS (Cat#: 20012-027, Thermo Fisher Scientific, USA) and transferred as frozen cell pellets to perform tandem mass tag (TMT)-based expression proteomics analysis as previously described.^99^ Cells were lysed with 500 μl of lysis buffer (8 M urea, 1% SDS and 50 mM Tris, pH 8.5, with protease and phosphatase inhibitors added) and sonicated to shear DNA aggregates. Once centrifugation was completed, the protein concentrations were measured by following a Micro BCA Protein Assay kit (Cat#: 23235, Thermo Fisher Scientific, USA). A total of 300 μg of protein was aliquoted from each sample and reduced with 5 mM DTT for 1 hour at room temperature, alkylated with 15 mM iodoacetamide for 1 hour at room temperature in the dark and then quenched with 10 mM DTT for 15 minutes at room temperature. Alkylated proteins were purified via chloroform-methanol precipitation,^100^ resuspended in denaturing buffer (8 M urea and 50 mM Tris, pH 8.5) and diluted with 7 volumes of 50 mM Tris, pH 8.5. Protein was digested using Trypsin/Lys-C mix (Cat#: A40009, Thermo Fisher Scientific, USA) in an enzyme:protein ratio of 1:25 and incubated overnight at 37°C. A second digestion was performed with an additional Trypsin/Lys-C mix (enzyme:protein ratio of 1:50) for 5 hours. Peptides from each sample were then desalted using a Water’s tC18 SepPak plate (Cat#: 186002321, Waters, USA), dried and resuspended in 100 μl of 0.1 M TEAB buffer, pH 8.5.

For each sample, 200 μg of peptides were labeled via TMT11plex Isobaric Label Reagent kit (Cat#: A34808, Thermo Fisher, USA) in TMT reagent:peptide ratio 4:1. Once the TMT labeling efficiency was confirmed by mass spectrometry analysis to be greater than 99%, the reaction was quenched with 0.5% with hydroxylamine for 15 minutes at room temperature. Equal amounts of each TMT-labeled sample were combined, desalted using Water’s tC18 SepPak plate (Cat#: 186002321, Waters, USA) and fractionated by HPLC using a Waters XBridge C18 column (3.5 μm, 300 × 4.6 mm) with gradient of 10–40% mobile phase B (90% acetonitrile with 5 mM ammonium formate, pH 10) in mobile phase A (5 mM ammonium formate with 2% acetonitrile). Final fractionated peptide material was pooled into 24 fractions (∼1-2 μg of peptides per fraction). Each fraction was analyzed using an Orbitrap Fusion Lumos mass spectrometer (Thermo Fisher Scientific, USA) equipped with a Reprosil-Pur column (1.9-μm beads, 75-μm ID × 15-μm tip × 20 cm, 120 Å). Samples were run using gradients of 6-28% mobile phase B (80% acetonitrile with 0.1% formic acid) in mobile phase A (0.1% formic acid) using the SPS MS3 mode.

Raw mass spectrometry data files were processed by Proteome Discoverer (PD) 2.4 (Thermo Fisher Scientific, USA). Searches were conducted using the PD Sequest node against the UniProt human canonical database (downloaded in July 2019 containing 21,482 sequences including common mass spec contaminants) with the following parameters: 10 ppm precursor tolerance, MS2 fragment ion tolerance of 0.6 Da, static modifications of TMT (+229.163 Da) on lysine and peptide N-terminal, and carbamidomethylation of cysteine residues (+57.021 Da), while oxidation of methionine residues (+15.995 Da) was set as a variable modification, and three missed cleavages were allowed. Peptide spectrum matches were filtered using SPSmatch > 60, precursor interference < 50, average TMT S/N > 10, and false discovery rate (FDR) determined by PD Percolator node < 1%. Shared peptides were used to assemble peptides to proteins and protein groups but were excluded from quantitative analysis. TMT intensities were corrected for isotopic impurities; Thermo Fisher Scientific provided a QC sheet specific to the TMT batch used in the analysis. The resulting table with TMT intensities was exported and processed with an internally developed R script (https://github.com/Novartis/px_tmt_daa) that filtered to protein FDR of 1% for each TMT plex, and normalized TMT intensities across four TMT11 plexes using CONSTANd procedure.^101^

Given the high biological variability among cell lines from different donors, instead of using mean protein abundance values across treatment groups (*n*=2), protein changes were visualized for individual donors, where cells derived from the same donor were used for both treatment and control conditions (Figure S5). Only proteins quantitated in both donors were used for analysis, with 7,632 of a total of 8,666 proteins quantitated in either donor. To evaluate protein dysregulation, R package clusterProfiler^102^ was used to carry out enrichment analysis with MetabaseMap (GeneGO, Clarivate, Philadelphia, PA) and MSigDB (Broad Institute, Cambridge, MA) geneset collections. UniProt primary accessions were converted to ENTREZ ID using the org.Hs.eg.db R package. Both up- and downregulated proteins in either donor were determined by selecting proteins outside of the confidence area (ellipse) at a level of 0.9999 as calculated by ggplot package (∼400 proteins). A list of all identified proteins was used as a “universe of possible proteins” to estimate the significance of enriched terms, and default cut-offs (pval < 0.01 and qval < 0.2) were applied to select significantly enriched gene sets.

The raw mass spectrometry proteomics data is available in ProteomeXchange Consortium via the PRIDE^103^ partner repository with the dataset identifier (to be included upon publication).

#### *In vivo* models

All studies described in this report were performed according to the UK Animals (Scientific Procedures) Act 1986.

##### Monocrotaline model

Animals (male Wistar Kyoto rats, Harlan UK) were injected with subcutaneous monocrotaline (Crotaline, Sigma, Poole, UK) at a dose of 60 mg/kg (0.5 ml/kg).^16^ On day 14, animals were dosed with 0.5 ml test agent or vehicle orally (p.o.) for 7 days either once or twice per day as indicated.

##### Sugen/hypoxia model

Animals (male Wistar Kyoto rats, Harlan UK) were injected with a subcutaneous Sugen (SU5416, Sigma, Poole, UK) at a dose of 20 mg/kg (1 ml/kg) before being caged in hypoxic conditions (10% O_2_, normobaric with constant humidity and CO_2_ levels, 24 hours/day) for 14 days.^16^ Animals were then returned to normoxia (21% O_2_) and test agents were administered via oral gavage (0.5 ml) once daily for a further 14 days. All SMURF1 inhibitors were prepared in 0.5% methyl cellulose, while 0.5% Tween 80 and imatinib were prepared in sterile water. At study end, animals were anesthetized using ketamine/medetomidine, the jugular vein was surgically exposed and blood flow was isolated with a distal ligature. A small hole was made in the vessel and a 2F Millar pressure/volume catheter was introduced and progressed into the right ventricle (RV), where an average RV pressure was measured during systole (RVSP). The lungs were excised from the rats and inflated with 10% neutral-buffered formalin and immersed in the same solution for 24–48 hours to complete fixation.

#### Histology

##### Histology for human specimens

Formalin-fixed, paraffin-embedded lung sections from 33 patients with PAH and 19 non-PAH patients undergoing transplant were obtained from approved tissue banks in Royal Papworth Hospital Research Tissue Bank, UK (Research ethics: 08/H0304/56+5) and Paris, France (CPP EST-III n°18.06.06, Le Kremlin-Bicêtre, France). All patients gave informed consent.

##### Dual immunohistochemistry on human specimens

A dual immunohistochemical assay was performed using the Ventana Discovery Ultra platform (Roche Diagnostics, USA). Sequential incubation and detection of primary antibodies were applied after heat-induced epitope retrieval at pH 8.5. First, Anti-vWF antibody (1:500 dilution, Cat#A0082, Dako) was added, followed by anti-rabbit-HRP conjugated secondary antibody (Omni Map anti-rabbit HRP, Cat# 460-4311, Roche Diagnostics, USA) and visualized by addition of DAB. Next, an anti-α-SMA antibody (1:16,000 dilution, Cat#A2547, Sigma) was added to the same slide and detected by adding of anti-mouse NP antibody and anti-NP AP conjugate and visualized by using the DISCOVERY Red kit (all reagents: Roche Diagnostics, USA). Slides were scanned using an Aperio Scanscope AT (Leica Biosystems, IL, USA).

##### Co-localization immunohistochemistry on human specimens

A bright field dual marker co-localization method was established to determine the localization of SMURF1 relative to α-SMA. Separately, co-localization studies for SMURF1 and vWF were performed on serial slides from the same tissue blocks, on human histology specimens using the Ventana Discovery Ultra platform (Roche Diagnostics, USA). Firstly, serial slides were stained for each of the individual markers. Sequential incubation and detection of primary antibodies were applied after heat-induced epitope retrieval at pH 8.5. SMURF1 detection was performed by administration of anti-SMURF1 antibody (1:800 dilution, Cat#AB57573, Abcam, USA), followed by incubation with anti-mouse HRP conjugated secondary antibody (Omni Map anti-mouse HRP Cat#760-150, Roche Diagnostics, USA) and visualized with purple chromogen (DISCOVERY Purple kit; Cat#760-229, Roche Diagnostics, USA). For vWF detection, anti-vWF antibody was applied (1:500 dilution, Cat#A0082, Agilent-Dako, USA) followed by anti-rabbit NP-conjugated antibody (Cat#760-4817, Roche Diagnostics, USA) and anti-NP antibody (Cat#760-4827, Roche Diagnostics, USA) and visualized by adding yellow chromogen (DISCOVERY Yellow kit; Cat#760-239, Roche Diagnostics, USA). For α-SMA detection, anti-α-SMA antibody (1:16,000 dilution, Cat#A2547, Sigma-Aldrich, USA) was added to the same slide and detected by addition of anti-mouse NP-conjugated secondary antibody (Cat#760-4816, Roche Diagnostics, USA), followed by addition of anti-NP antibody (Cat#760-4827, Roche Diagnostics, USA) and visualized by administration of yellow chromogen (DISCOVERY Yellow kit; Cat#760-239, Roche Diagnostics, USA). Next, serial sections were co-stained for SMURF1+vWF and separate serial slides were co-stained for SMURF1+α-SMA using the same antibody and protocols described for the single stains. Specificity of staining was determined using isotype controls applied to serial sections from selected blocks: for SMURF1 (1:800 dilution, isotype mouse IgG2a Cat#70-4724, Tonbo Biosciences, USA), α-SMA (1:1430 dilution, isotype mouse IgG2a Cat#70-4724, Tonbo Biosciences, USA) and vWF (1:1760 dilution, isotype rabbit IgG Cat#I-1000, Vector Labs, USA). Co-localization assays required denaturation before the incubation of the second primary antibody to prevent non-specific cross-reactivity. Slides were scanned using an Aperio Scanscope AT. Where two antibodies co-localize, a color shift to red is evident.

##### Immunohistochemistry on rodent specimens

*Monocrotaline model*. FFPE slides were dual immunostained using the Ventana Discovery Ultra platform (Roche Diagnostics, USA). Sections were dewaxed, and antigens were retrieved using CC1 (pH 8.5) and incubated for 16 minutes. Dual immunostaining was performed by sequential incubation of the same slide with an anti-vWF antibody (1:500 dilution, Cat#A0082, Agilent-Dako, USA) followed by its detection with anti-rabbit HRP-conjugated secondary antibody (Rabbit Omni Map HRP, Cat#460-4311, Roche Diagnostics, USA) and visualized using ChromoMAP DAB kit (Cat#760-159 Roche Diagnostics, USA). Thereafter, an anti-α-SMA antibody was applied to the same slide (1:16,000 dilution, Cat#A5228, Sigma-Aldrich, USA) and detected with anti-mouse NP-conjugated secondary antibody (Cat# 760-4816, Roche Diagnostics, USA) and visualized using DISCOVERY Red kit (Cat# 760-228, Roche Diagnostics, USA). The specificity of staining was confirmed by the absence of staining on slides from representative blocks with the same isotype controls at the same dilutions as used for human sections described above. Slides were scanned using an Aperio XT slide scanner. Arteriole remodeling following monocrotaline exposure was measured by counting 100 small vessels (< 100 μm diameter) and assigning each vessel as either non-muscularized (no α-SMA staining), partially muscularized or fully muscularized (thick unbroken wall of smooth muscle), and then the percentage distribution of each was calculated per group. The histological analyst was blind to the experimental conditions.

*Sugen/hypoxia model*. Lungs were processed and dual α-SMA/vWF immunohistochemistry was performed as described for the monocrotaline model. Arteriole remodeling was measured by image analysis software (ImagePro, Media Cybernetics, UK), which quantified the α-SMA positive pixels as a measure of percent muscularization of selected vessels < 100 μm in diameter.

*Phosphorylated (p)SMAD1/5/8*. Staining was performed using the Ventana Discovery Ultra platform (Roche Diagnostics, USA) as described above. Phospho-SMAD1/5/8 was detected by incubation with an anti-pSMAD1/5/8 antibody (1:50 dilution, Cat#9511, Cell Signaling Technologies, USA), followed by incubation with HRP-conjugated polyclonal swine anti-rabbit secondary antibody (1:200, Cat#E03553, Dako-Agilent, USA).

#### *In silico* screening for E6AP allosteric inhibitors

##### Remodeling allosteric inhibited E6AP

HHblits was used to identify up to 500 homolog proteins for the HECT domain of E6AP and SMURF1 in the uniref30 database. Redundant sequences with an identity higher than 90% were removed using mmseq2 and the obtained profiles were aligned with MAFFT.^104^ The protein alignment was manually inspected and adjusted to ensure that conserved residues were aligned correctly. This alignment was used to instruct modeler^105^ to build a structural model of E6AP based on the inhibited conformation of SMURF1. To remove any steric clashes from the model, Prime energy minimization was used with the OPLS-4 force field and VGSB solvation model.^106^^,^^107^ Minimization was performed in iteration, each with 65 steps, or until converging to a 0.01 kcal/mol/Å gradient.

##### Ligand preparation and virtual screening

A total of ∼8 million commercially available, drug-like molecules were downloaded from the ZINC database. The ligands were prepared using Ligprep (Schrödinger LLC, NY). Stable protonation and tautomerization states at 7.0±2 pH were generated for each molecule by Epik.^108^ Chirality was maintained as listed in ZINC. Virtual screening was performed in two phases using Glide molecular docking.^109^ First, the entire compound library, including all generated isoforms, was docked using the low-accuracy, HTVS docking mode. Second, the top-ranking 10^th^ percentile molecules were re-docked using the more rigorous SP docking mode. The top-ranking 1000 ligands from the second phase of the docking were visually inspected and, based on their binding pose, 32 compounds were selected, purchased and used for experimental verification in the E6AP split-CAT *E. coli* system.

### Quantification and statistical analysis

#### Cell and animal studies

Data are represented as mean+/-SEM. Normality of data distribution was assessed by Kolmogorov-Smirnov test and differences between groups were evaluated as described. Statistical analysis was performed as stated in Prism 10.3.1 (464) for Macintosh (GraphPad Software).

#### Proteomic studies

Analysis was undertaken as described, with code provided at https://github.com/Novartis/px_tmt_daa.

## Supplemental information

Table S1. Alignment of multiple HECT E3 ligases showing the conserved glycine residue at the hinge connecting the N- and C-lobes, related to Figure 1D The conserved Gs (glycine 634 of SMURF1; first line) are colored red. The sequence of yeast HECT E3-ligase Rsp5 was used as a probe for BLAST and retrieved 5,000 HECT sequences from the “non redundant protein sequences (nr)” at NCBI. The proteins were aligned by fast-Fourier-transform-based multiple alignment MAFFT v7.158b (2014/06/27, http://mafft.cbrc.jp/alignment/software/ MBE) 30:772-780 (2013), NAR 30:3059-3066 (2002). Full table of 5,000 included as supplemental pdf file (Figure S5).

Table S2. Summary of histopathological evaluation of SMURF1 inhibitor Cpd-6-treated MCT rats, related to Figure 5 Organs were harvested and processed for immunohistochemistry and scored by a blinded, trained pathologist. Findings were scored as follows: Grade 1 = minimal (very few/very small); Grade 2 = slight (few/small); Grade 3 = moderate (moderate number/ moderate size). Dash (-) indicates organ examined, no pathologic finding noted. Multiple animals per treatment group were assessed (*n* as indicated). Data are mean grade per tissue.
