# Supplementary material for: Therapeutic potential of allosteric HECT E3 ligase inhibition
Source: Cell. 2025 May 15;188(10):2603–2620.e18. doi: 10.1016/j.cell.2025.03.001 (PMC12087876; doi:10.1016/j.cell.2025.03.001)
Supplement: Table S2. Summary of histopathological evaluation of SMURF1 inhibitor Cpd-6-treated MCT rats, related to Figure 5 — Organs were harvested and processed for immunohistochemistry and scored by a blinded, trained pathologist. Findings were scored as follows: Grade 1 = minimal (very few/very small); Grade 2 = slight (few/small); Grade 3 = moderate (moderate number/ moderate size). Dash (-) indicates organ examined, no pathologic finding noted. Multiple animals per treatment group were assessed (n as indicated). Data are mean grade per tissue. [file mmc2.pdf]

Organ & findings	MCT-Veh *n*=5	1mg/kg *n*=4	10mg/kg *n*=4	30mg/kg *n*=4	100mg/kg *n*=4
**Heart: inflammation**	1.0	1.0	1.0	1.0	1.0
**Lungs: inflammation**	1.3	1.0	1.0	1.0	1.3
**Lungs: foam cells, alveolar**	1.0	1.0	1.0	1.0	1.0
**Stomach: dilat. Mucosal gland**	1.0	-	-	-	-
**Small intestine**	-	-	-	-	-
**Large intestine**	-	-	-	-	-
**Adrenal glands: vacuolation**	-	-	1.0	1.0	1.0
**Eyes**	-	-	-	-	-
**Femur**	-	-	-	-	-
**Spleen**	-	-	-	-	-
**Knee joint**	-	-	-	-	-
**Sternum**	-	-	-	-	-
